# Nrf2 and HIF1α converge to arsenic-induced metabolic reprogramming and the formation of the cancer stem-like cells

**DOI:** 10.7150/thno.42903

**Published:** 2020-03-04

**Authors:** Zhuoyue Bi, Qian Zhang, Yao Fu, Priya Wadgaonkar, Wenxuan Zhang, Bandar Almutairy, Liping Xu, M'Kya Rice, Yiran Qiu, Chitra Thakur, Fei Chen

**Affiliations:** 1 Department of Pharmaceutical Sciences, Eugene Applebaum College of Pharmacy and Health Sciences, Wayne State University, 259 Mack Avenue, Detroit, MI 48201, USA; 2 School of Health Sciences, Wuhan University, 115 Donghu Road, Wuhan 430071, China; 3 Hubei Provincial Key Laboratory of Applied Toxicology, Hubei Provincial Center for Disease Control and Prevention, 8 Zhudaoquanbei Road, Wuhan 430079, China; 4 College of Pharmacy, Al-Dawadmi Campus, Shaqra University, P.O.Box 11961, Riyadh, Kingdom of Saudi Arabia.

**Keywords:** arsenic, Nrf2, HIF1α, metabolic reprogramming, cancer stem cells

## Abstract

In this report, we demonstrated that inorganic arsenic (iAs) induces generation of the cancer stem-like cells (CSCs) through Nrf2-dependent HIF1α activation, and the subsequent metabolic reprogramming from mitochondrial oxidative phosphorylation to glycolysis in epithelial cells.

**Methods**: Genome-wide ChIP-seq analysis was performed to investigate the global binding of Nrf2 and/or HIF1α on the genome in the cells treated with iAs. Both untargeted metabolomics and UDP-^13^C-glucose flux were applied to determine metabolic reprogramming in the iAs-induced CSCs. The role of Nrf2 on iAs-induced HIF1α and other stemness gene expression was validated by lentiviral transfection of Nrf2 inhibitor Keap1 and CRISPR-Cas9-mediated Nrf2 gene knockout, respectively.

**Results**: The CSCs induced by iAs exhibit a diminished mitochondrial oxidative phosphorylation and an enhanced glycolysis that is actively shunted to the hexosamine biosynthetic pathway (HBP) and serine/glycine pathway. ChIP-seq data revealed that treatment of the cells with iAs amplified Nrf2 enrichment peaks in intergenic region, promoter and gene body. In contrast, a shift of the HIF1α peaks from distal intergenic region to gene promoter and the first exon was noted. Both Nrf2 and HIF1α are responsible for the iAs-induced expression of the glycolytic genes and the genes important for the stemness of the CSCs. Intriguingly, we also discovered a mutual transcriptional regulation between Nrf2 and HIF1α. Inhibition of Nrf2 by lentiviral infection of Keap1, or knockout of Nrf2 by CRISPR-Cas9 gene editing, not only blocked iAs-induced HIF1α activation, but reduced the expression of the key stemness genes for the formation of CSCs also.

**Conclusion**: We demonstrated that Nrf2 activation is an initiating signal for iAs-induced HIF1α activation, and Nrf2 and HIF1α played a concerted role on inducing metabolic reprogramming and the CSCs.

## Introduction

As one of the most abundant environmental carcinogens worldwide, arsenic affects about 200 million people in more than 70 countries, including the United States 1. The main sources of environmental arsenic, especially the inorganic trivalent arsenic (iAs), include groundwater contamination, working place air pollution, and application of certain pesticides in agricultural activities 2. The greatest threat to public health comes from the direct consumption of groundwater that flows through rocks and minerals containing naturally deposited arsenic. In recent years, an emerging concern of environmental arsenic exposure is the findings that some food crops are highly capable of enriching arsenic in the grain or other edible parts from the iAs-contaminated irrigation water and soil, such as rice, fruits and certain vegetables 3. Extensive studies had been made in the past on how iAs induces human cancers in lung, skin, bladder, liver, kidney, hematopoietic systems, and other organs. Through mimicking the environmental iAs exposure, we previously showed that consecutive treatment of the human bronchial epithelial cells (BEAS-2B) with 0.125 to 0.25 μM iAs (~9 to 18 ppb) for six months, induced generation of the cancer stem-like cells (CSCs) 4. Intriguingly, extensive studies unraveled that iAs is a poorer mutagen in mammalian cells. Thus, the cancer causing effect of iAs may be achieved through its co-carcinogen or epigenetic regulations.

Few reports had suggested that iAs can convert normal stem cells to CSCs through malignant transformation 5-7. However, it is largely unknown whether iAs can induce generation of the CSCs from the normal differentiated cells until our studies showing generation of CSCs through consecutive iAs treatment of the non-cancerous human bronchial epithelial cells 4. Similar to the normal stem cells, the iAs-induced CSCs also exhibited increased expression of Oct4, Sox2, Klf4, Myc, and several other stemness transcription factors. In contrast, genes important for mitochondrial oxidative phosphorylation (OXPHOS), tricarboxylic acid (TCA) cycle, and other mitochondrial function are substantially down-regulated, indicative of metabolic reprogramming. In addition to the tumorigenicity in mice, these CSCs also showed characteristics of self-renewal *in vitro* and *in vivo*, and resistance to chemodrug-induced apoptosis.

Nrf2 was traditionally viewed as a master regulator of the cellular antioxidant response. Emerging evidence, however, suggested that Nrf2 is also a key driving factor for tissue fibrosis, cancer progression, tumor cell metastasis, and therapeutic resistance of cancers 8-10. Meanwhile, Nrf2 is able to induce expression of several genes contributing to the stemness of the stem cells, such as ALDH 11 and Notch1 12. The hypoxia inducible factor 1α, (HIF1α), on the other hand, is capable of inducing human embryonic stem cell (hESC)-like transcriptional program in cancer cells. Silencing HIF1α decreased the expression of Oct4, Sox2 and Nanog, the central stemness genes in cancer cells 13. Both Nrf2 and HIF1α are capable of enhancing glycolysis of the cells. There are reports showing activation of Nrf2 and HIF1α by iAs 14, 15, respectively. However, it is unknown whether iAs-induced malignant transformation and the formation of CSCs requires either Nrf2 or HIF1α, or both. It is also unknown whether Nrf2 and HIF1α are two independent signaling pathways or concerted together through cross-talks in the iAs-induced metabolic reprogramming during the formation of the CSCs. For Nrf2 and HIF1α themselves, the mechanisms of their activation by iAs remain to be fully elucidated.

In the present study, we provide evidence showing that iAs induced a Nrf2-dependent activation of HIF1α, which in turn concerted with Nrf2 to promote metabolic shift from mitochondrial OXPHOS to glycolysis, most notably, in the hexosamine biosynthesis pathway (HBP) and the serine/glycine pathway, the most important side pathways of glycolysis. In addition, we showed that both Nrf2 and HIF1α can participate in the transcriptional regulation of the key genes important for malignant transformation and the generation of the CSCs.

## Materials and Methods

### Cell culture

The human bronchial epithelial cell line BEAS-2B were purchased from the American Type Culture Collection (ATCC, Manassas, VA). The cells were cultured in DMEM medium (Invitrogen, Grand Island, NY) with 5% fetal bovine serum (Invitrogen, Grand Island, NY), 1% penicillin-streptomycin (Sigma, St. Louis, MO) and 1% L-Glutamine, and maintained in humidified incubator at 37 °C with 5% CO_2_.

### Western blotting

BEAS-2B cells were seeded in a 6-well plate (3.5×10^5^ cells per well) and treated with various concentrations of iAs for the indicated times. Cells were lysed by 1×RIPA cell lysis buffer (Cell signaling) supplemented with protease and phosphatase inhibitors cocktail (Roche, Indianapolis, IN) and 1mM PMSF. Collected cell lysates were then homogenized by sonication and insoluble debris was removed through centrifugation of 12,500g at 4 °C for 15 minutes. The concentrations of protein were then determined using Pierce BCA Protein Assay Kit (Thermo Scientific, Rockford, IL). The protein samples were prepared using 4 × LDS sample buffer (Invitrogen) with dithiothreitol at a final concentration of 200 mM and were denatured by boiling at 95°C for 5 minutes before separation by SDS-PAGE gel. Separated samples were then transferred onto PVDF membrane (Invitrogen) and blocked with 5% non-fat milk diluted in TBST for 1 hour at room temperature. After extensive washing with TBST, the membranes were incubated with the indicated primary antibodies for overnight at 4°C and corresponding HRP-linked second antibodies for 1 hour at room temperature before detecting. ECL substrates (Thermo Scientific, Millipore and Westpico Plus) were used to visualize the signals on autoradiography films. Primary antibodies against Sox2, Klf4, C-myc, Nrf2, HIF1α, Perk, JNK, pJNK, TBC1D7C, Actin, GAPDH and HRP-linked mouse and rabbit IgG were purchased from Cell Signaling technology. Antibodies against Keap1 and BACH1 were purchased from Santa Cruz technology. Antibody against Grp78 was purchased from Abcam.

### Luciferase reporter gene activity assay

To measure the activities of Nrf2 and HIF1α, BEAS-2B cells were incubated in75-cm^2^ flasks (1.5×10^6^ cells per flask) and then treated with 1μM iAs at various time points. Cells were harvested after treatment, and the nuclear extract lysates were obtained by using the Nuclear Extraction Kit (ab113474, Abcam), according to the manufacturer's protocols. The activities of Nrf2 and HIF1α in nuclear extract lysates were detected using the Nrf2 (ab207224, Abcam) and HIF1α (ab133104, Abcam) Transcription Factor Assay Kit, respectively.

### Chromatin immunoprecipitation-sequencing (ChIP-seq)

For ChIP-seq, ten million cells were fixed and subjected to immunoprecipitation using ChIP-grade antibodies against Nrf2 and HIF1α from Active Motif (Carlsbad, CA). The procedures of ChIP, preparation of input and control DNA, DNA sequencing, and data analysis were performed as what we had recently reported 16. All ChiP-seq data can be accessed at https://www.ncbi.nih.gov/geo/query/acc.cgi?acc=GSE145834

### Untargeted global metabolomics

Six replicates of each control and treatment conditions containing 4 × 10^6^ cells, including the iAs-induced CSCs, were prepared and subjected to LCMS and GCMS detections for metabolites in glycolysis, HBP and serine/glycine shunt, TCA, nucleotide pools, amino acids, peptides, lipids, and xenobiotics, as guided by Metabolon Inc. (Research Triangle Park, NC). LCMS detection includes a one step liquid-liquid organic solvent extraction of cultured cells and separation on a 1mm × 150mm HILIC specific column in a 35 min cycle. GCMS detection includes a one step liquid-liquid extraction followed by MTBSTFA derivatizations.

### UDP-^13^C Glucose flux

Three replicates for each treatment conditions containing 4 × 10^6^ cells in 10cm dishes were exposed with 45mg/L UDP-^13^C glucose DMEM medium for 1h labeling. After labeling, the medium was removed and the cells were washed with cold 150mM ammonium acetate that was removed within 30 seconds. The cells were quenched by liquid nitrogen on the plates and allow evaporation until the majority of liquid nitrogen is eliminated. The prepared samples were wrapped in aluminum foil and stored at -80 ^o^C until for LCMS detection for UDP-^13^C glucose tracing that was performed by the University of Michigan Metabolomics Core.

### Lentiviral transfection

Human Keap1 overexpression clone lentiviral particle (RC202189L4V) and Lentiviral control particle (PS100093V) were purchased from Origene Technologies Inc.

BEAS-2B cells were seeded in 24-wells plate (5 × 10^4^ cells per well) and incubated for 20 hours, and then infected with Keap1 lentiviral overexpression particle and Lentiviral control particle for 20 hours, respectively. To improve the efficiency of infection, 8μg/ml polybrene was added. Stably transfected clone were established by addition of puromycin (1μg/ml) (Invitrogen, Grand Island, NY) for 14 days.

### CRISPR-Cas9 knockout of Nrf2

CRISPR-Cas9 gene editing for Nrf2 knockout was performed following the protocols we recently reported for mdig gene knockout 16. Briefly, the Nrf2 coding sequence was inputted into online design tool, CRISPOR (http://crispor.tefor.net/), to design single guide RNA (sgRNA). Two sgRNAs targeting the exon 2 of human Nrf2 gene with high score were chosen: sgRNA-1 (5'-TATTTGACTTCAGTCAGCGA-3'); sgRNA-2 (5'-GCGACGGAAAGAGTATGAGC-3'). Single-stranded sense and antisense primers of each sgRNA were annealed to form double-strand sgRNA by heating at 95 ^o^C for 5 min, and then cooling down to room temperature at 5 ^o^C/min. Each double-strand sgRNA were ligated with Bpil (Bpsl)-digested vector, pSpCas9-2A-Blast, by T4 ligase (Thermo Fisher Scientific) for 10 min at room temperature. The ligation products were transfected into DH5α Competent Cells (Invitrogen) according to the manufacturer's protocol. Constructs were validated by Sanger sequencing using U6 promoter primer.

### Plasmid transfection and blasticidin selection

BEAS-2B cells (2.5 × 10^5^) were seeded in 6-well plates for overnight, and then transfected with CRISPR-Cas9 vectors by Lipofectamine 2000 (Invitrogen) according to manufacturer's protocol. Forty-eight hours after the transfection, cells were split into 10 cm dishes. After twenty-four hours, cells were cultured in growth medium with 4ug/ml of Blasticidin (Thermo Fisher Scientific) until the formation of single colonies. About 20 colonies from each sgRNA transfection were collected for western blotting to check Nrf2 expression using Cell Signaling D1Z9C XP rabbit anti-Nrf2 mAb. Colonies with the depletion of Nrf2 protein expression were designated as knockout cells (KO), and colonies with Nrf2 protein expression were designated as wild type cells (WT).

### Statistics

Student T-test was used to determine the statistical significance of quantitative data. For comparative analysis of ChIP-seq, standard normalization was achieved by down-sampling the usable number of tags for each sample in a group to the level of the sample in the group with the fewest number of tags. Enrichment peaks of Nrf2 and HIF1α were called using either the MACS^a^ or SICER^b^ algorithms. Metabolomics data were normalized to the total cellular proteins with a CV of 15%.

## Results

### iAs treatment enriches stemness genes

**Figure 1:**
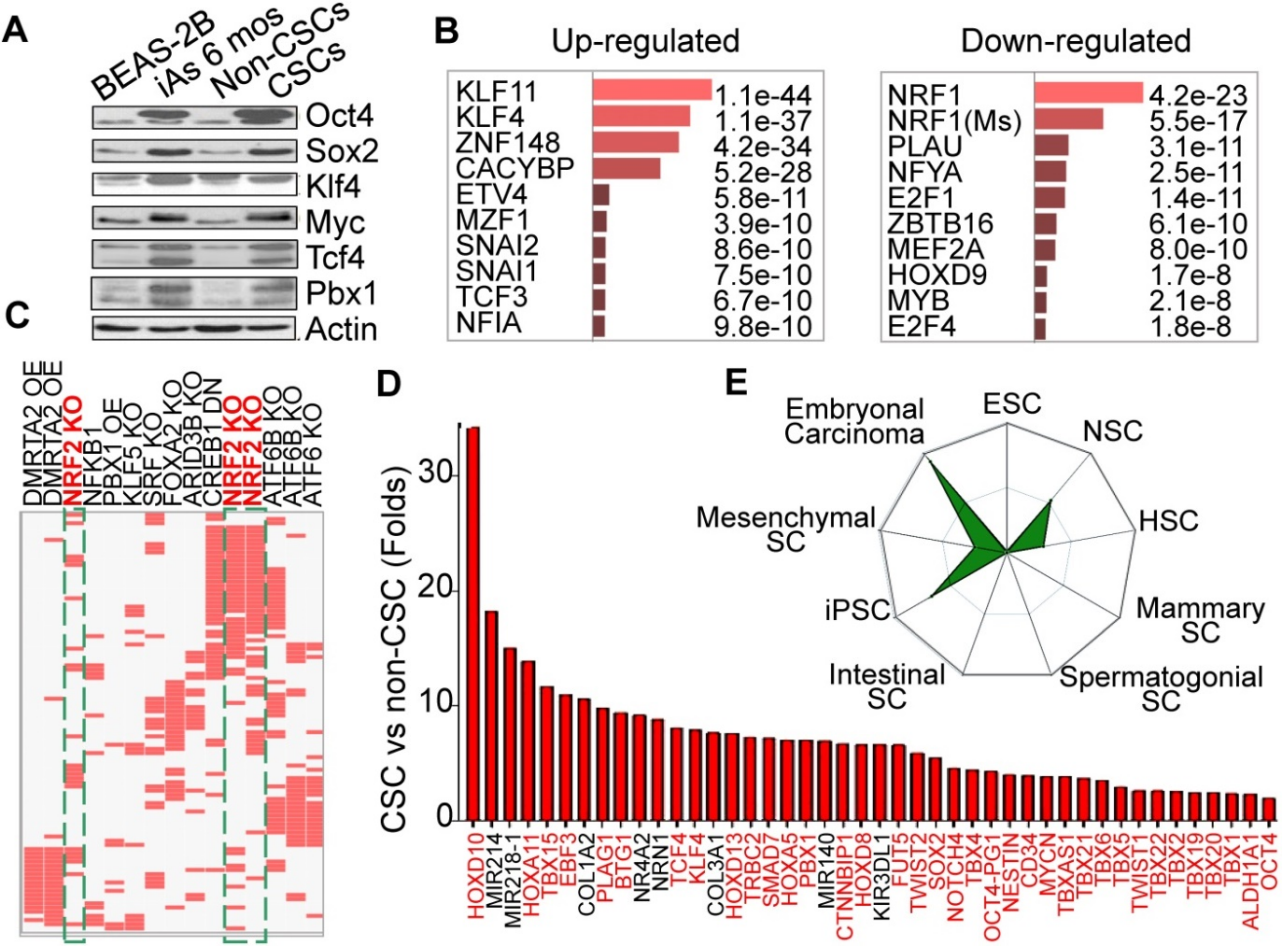
Consecutive iAs treatment induces CSCs. **A.** Increased stemness gene expression in the cells treated with 0.25 μM iAs for 6 months (iAs 6 mos) and the CSCs isolated from the iAs 6-month-treated cell population. **B.** Analysis of the genes that showed more than 2-fold differential expression between non-CSCs and CSCs by TRANSFAC and JASPAR PWMs programs. **C.** TF Perturbations assay of the up-regulated genes in CSCs. OE: overexpression; KO: knockout; DN: dominant negative/down-regulation. **D.** Relative expression levels of the indicated stemness genes in iAs-induced CSCs. **E.** Stem cell signatures of the up-regulated genes in CSCs.

Our earlier studies showed that consecutive treatment of the bronchial epithelial cells (BEAS-2B) with an environmentally relevant concentration of iAs, 0.125 - 0.25 μM ( ~9 - 18 ppb), for 4 - 6 months induced malignant transformation of the cells 4, some of which acquired characteristics of the CSCs, such as tumor sphere formation *in vitro* and tumorigenesis in nude mice. Westernblotting revealed that both iAs-transformed (iAs-6 mos) cells and the identified CSCs as we had reported previously 4 expressed higher levels of c-Myc, Oct4, Sox2, and Klf4 under the basal condition (Fig. 1A). Previous transcriptome assay suggested up-regulation of the stemness and Wnt signaling genes, and down- regulation of the genes for DNA repair and mitochondrial OXPHOS in these iAs-induced CSCs 4. To further characterize these iAs-induced CSCs, we re-analyzed these genes that had a more than 2-fold differential expression between BEAS-2B and CSCs by Enrichr programs TRANSFAC and JASPAR PWMs. For this analysis, the 5kb proximal promoter regions of these genes were scanned for statistical enrichment of conserved human transcription factor (TF) binding sites. The binding motifs of several stemness TFs, including KLF11, KLF4, SNAI1, SNAI2, TCF3, etc, were highly enriched in the promoters of these up-regulated genes in CSCs (Fig. 1B). This finding suggests that corresponding binding of these TFs might regulate those up-regulated genes in the iAs-induced CSCs. In contrast, the down-regulated genes are mostly regulated by the TFs for mitochondrial function and differentiation, such as NRF1, NFYA, MYB, HOXD9, etc. NRF1 is one of the most important transcription factors for mitochondrial DNA transcription and replication 17. An additional analysis using data sets of TF Perturbations Followed by Expression demonstrated three Nrf2 entries in the top 20-ranked TFs for the up-regulated genes (Fig. 1C), indicating that a substantial number of genes are regulated by the Nrf2 transcription factors in CSCs. Meanwhile, this analysis also revealed some MYC-regulated genes enriched in CSCs (data not shown), which is in agreement with the finding of increased expression of Myc in iAs-induced CSCs (Fig. 1A). Among the up-regulated genes, we indeed noted that more than 50 well-classified stemness genes are over-represented, such as Tbx family members, Tcf4, Klf4, Pbx1, Mycn, Twist2, Sox2, etc. (Fig. 1D). Using StemChecker software, we found that the gene expression pattern of the iAs-induced CSCs is highly similar to the induced pluripotent stem cells (iPSC), embryonal carcinoma, neuronal stem cells (NSC), and hematopoietic stem cells (HSC), suggesting that the iAs-induced CSCs are developmentally or hierarchically close to the adult stem cells, progenitor cells and/or embryonal carcinoma (Fig. 1E).

### Metabolic reprogramming in the iAs-induced CSCs

**Figure 2:**
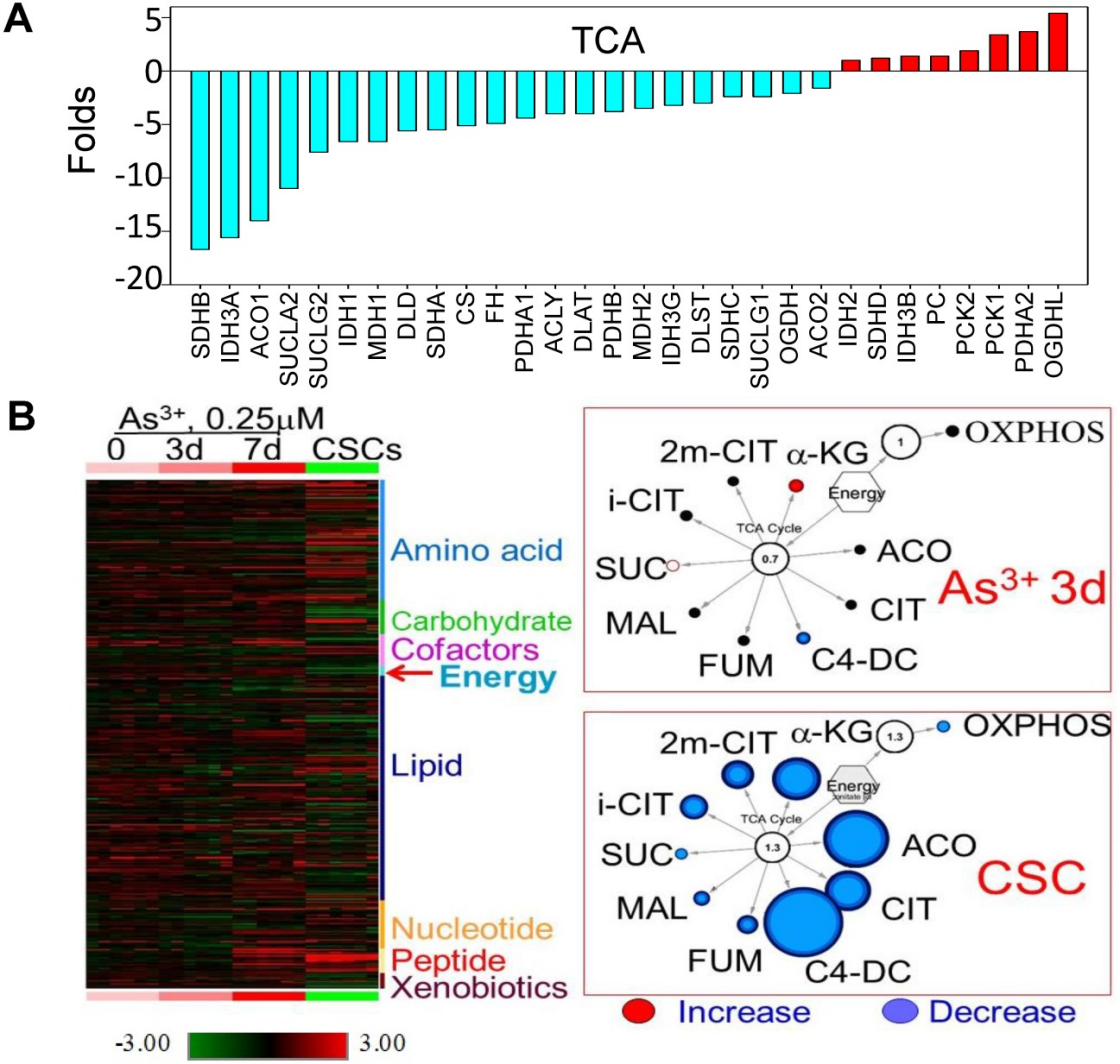
Diminished mitochondrial TCA cycle in the iAs-induced CSCs. **A.** The expression levels of the genes in TCA cycle. **B.** Metabolomics analysis of the BEAS-2B cells treated with 0.25 μM iAs for 3 days, 7 days, and 6 months (CSCs). Left panel shows heatmap of metabolites. Right two panels show decreased TCA cycle metabolites in the cells treated with iAs for 3 day and the iAs-induced CSCs.

**Figure 3:**
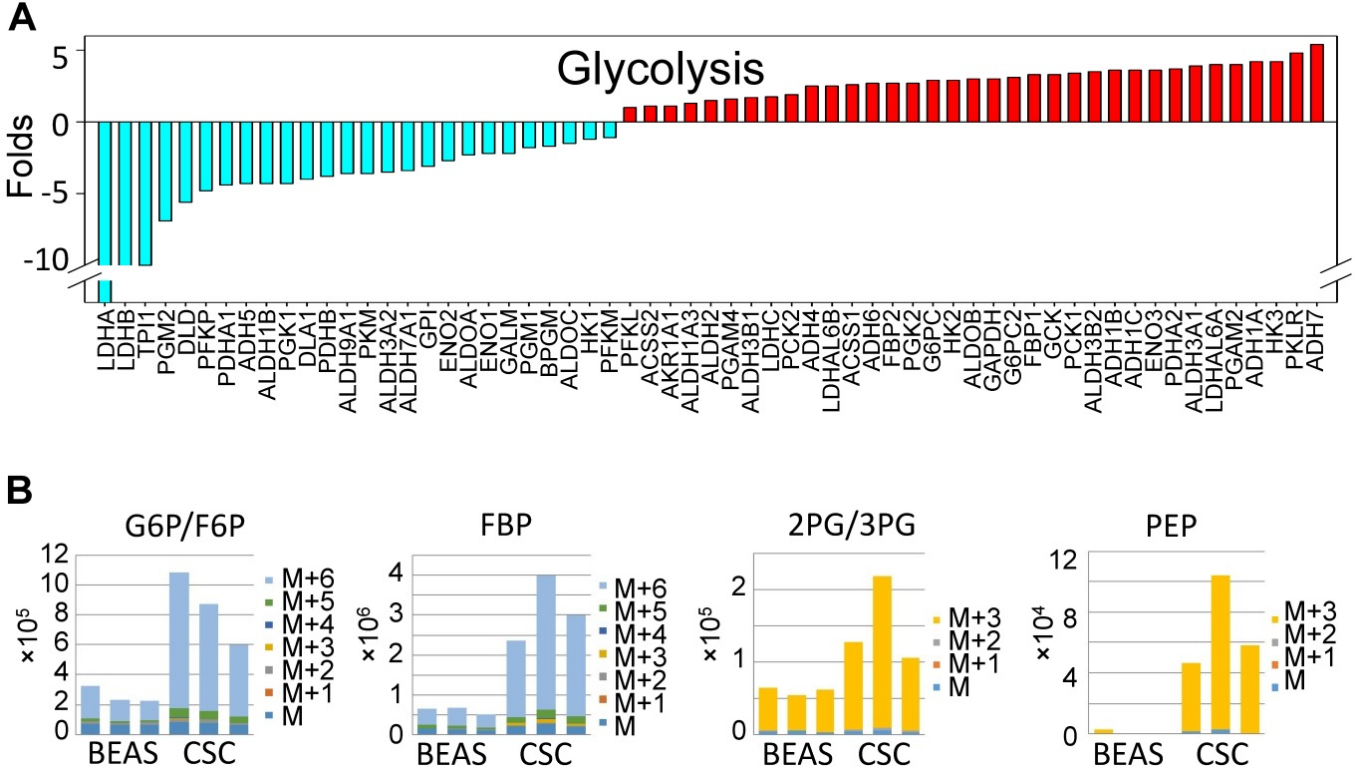
Up-regulation of glycolytic metabolism in the iAs-induced CSCs.** A.** The expression levels of the genes in major glycolytic pathways.** B.** UDP-^13^C-glucose tracing of the major glycolytic metabolites.

Decreased expression of the genes that are regulated by NRF1 suggested an impaired function of the mitochondria in the iAs-induced CSCs. Indeed, WikiPathway gene set enrichment analysis showed that mitochondrial OXPHOS and TCA cycle are the two of the top 20-ranked pathways of these down-regulated genes (sFig. 1A). Among 54 genes involved in OXPHOS, we found that 45 genes that were down-regulated for more than 2 folds in these CSCs cells relative to the control cells (sFig. 1B). For those genes listed as TCA cycle genes in the KEGG pathway, except OGDHL, PCKs, and few others, more than 70% of these genes are down-regulated in CSCs (Fig. 2A). In contrast, most of the genes in the glycolytic pathway are up-regulated in CSCs (Fig. 3A). This unique gene expression pattern implied that CSCs might feature a metabolic shift from mitochondrial TCA cycle to glycolysis. To confirm this, we first performed untargeted global metabolomics analysis between the control cells and iAs-induced CSCs. By using the DiscoveryHD4 platform for the maximum coverage of the metabolites, we detected significant changes of 570 metabolites in amino acid, carbohydrate, cofactors and vitamins, energy, lipid, nucleotide, peptide, and xenobiotics in the cells treated with 0.25 μM iAs for 3 days, 7 days, or 6 months (CSCs) (Fig. 2B). A pronounced reduction of all of the metabolites in TCA cycle, including citrate, isocitrate, α-ketoglutarate, succinate, fumarate, and malate, was noted in the CSCs. Even for a short-term treatment for 3 days, iAs induced a detectable decrease of these TCA cycle metabolites, except α-ketoglutarate (Fig. 2B).

To gain more detailed insights into the metabolic reprogramming in the iAs-induced CSCs, we cultured the cells with UDP-^13^C glucose and quantified isotope incorporation in metabolites of glycolysis. This assay revealed significant increases in flux from glucose into the glycolytic intermediates in CSCs. Quantifying the abundance of specific isotopologs generated via glycolysis suggested a much higher yielding of M6 glucose-6-phosphate/fructose-6-phosphate (G6P/ F6P), M6 fructose-bisphosphate (FBP), M3 phosphoenolpyruvate (PEP), M3 2-phosphoglycerate/3-phosphoglycerate (2PG/3PG), M5 ribulose 5-phosphate/ xylulose 5-phosphate (R5P/X5P), and others in CSCs (Fig. 3B and data not shown). M6 and M3 mean each metabolite labeled with six and three ^13^C atoms from UDP-^13^C glucose, respectively. These data clearly demonstrated that the iAs-induced CSCs rewired glucose metabolism into glycolysis or the Warburg effect.

### iAs induces Nrf2-dependent HIF1α activation

**Figure 4:**
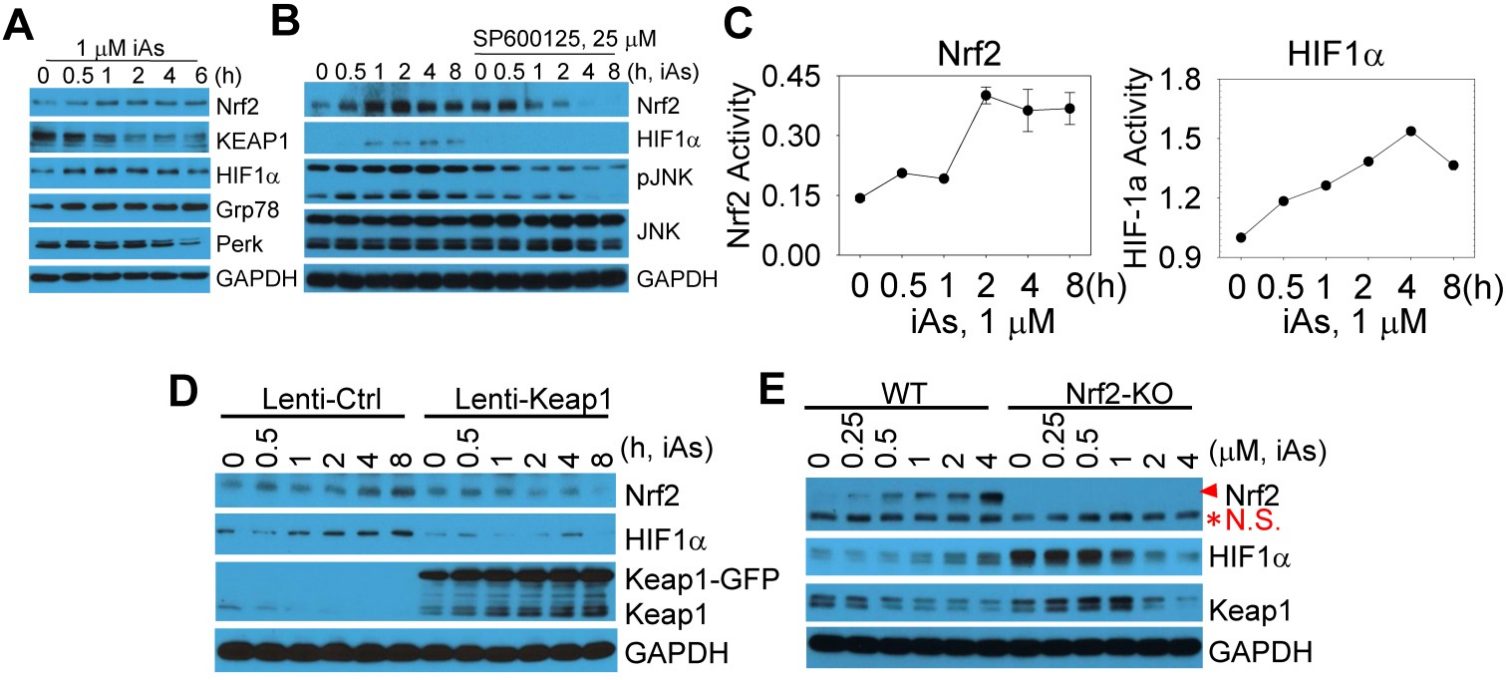
Activation of Nrf2 and Nrf2-dependent HIF1α expression in the cells treated by iAs. **A.** Time-dependent activation of Nrf2 and HIF1α. **B.** Involvement of JNK in iAs-induced Nrf2 as well as HIF1α. **C.** Luciferase reporter gene activities of Nrf2 and HIF1α in the cells treated with 1 μM iAs for the indicated times. **D.** Lentiviral transfection of Keap1 inhibited iAs-induced Nrf2 activation and HIF1α expression. **E.** Knockout of Nrf2 by CRISPR-Cas9 gene editing prevented HIF1α induction by iAs. Red asterisk denotes the non-specific (N.S.) band.

The above data indicated a metabolic shift from mitochondrial TCA cycle to glycolysis, which might be the key driving force for the generation of the CSCs induced by a sustained treatment of iAs. Also gene profiling through TF Perturbation revealed clustering of Nrf2 regulation of the upregulated genes in CSCs. Furthermore, both Nrf2 and HIF1α had been linked to metabolic reprogramming and the properties of stem cells 8, 13. Accordingly, we next determined if Nrf2 and HIF1α are activated by treating the BEAS-2B cells with 1 μM iAs for 0.5 to 6 h. A time-dependent activation of Nrf2 and HIF1α was observed, along with a time-dependent decrease of Keap1, an endogenous Nrf2 inhibitor that binds to and promotes Nrf2 ubiquitination and degradation (Fig. 4A). In addition, iAs is also capable of inducing Grp78 and Perk (Fig. 4A), two key components in the endoplasmic reticulum (ER) stress response pathway that is involved in Nrf2 activation 18. Earlier studies had suggested a limited effect of JNK on Nrf2 activation, despite JNK is able to phosphorylate Nrf2 19. In the case of iAs-induced Nrf2 activation, we found that inhibition of JNK by specific JNK inhibitor, SP600125, prevented Nrf2 and HIF1α activation induced by iAs significantly (Fig. 4B), indicating that the involvement of JNK in iAs-induced Nrf2 signaling may through mechanisms other than Nrf2 phosphorylation, such as affecting the phosphorylation and/or stability of Keap1, VCP (p97) and SQSTM1 that contribute to Nrf2 activation 20. To additionally validate activation of Nrf2 and HIF1α by iAs, we also quantified the luciferase reporter gene activities of both Nrf2 and HIF1α. The peak activation of Nrf2 and HIF1α occurred at 2h and 4h of 1 μM iAs treatment, respectively, indicating that HIF1α activation by iAs is possibly Nrf2 dependent (Fig. 4C). To confirm this, we inhibited Nrf2 by lentiviral-based overexpression of Keap1, an endogenous inhibitor for Nrf2, and disrupted Nrf2 gene through CRISPR-Cas9 gene editing (sFig. 2), respectively. As indicated in Figs. 4D, inhibition of Nrf2 prevented iAs-induced HIF1α accumulation significantly. In Nrf2 knockout (KO) cells, although the basal level of HIF1α was higher in the KO cells relative to the wild type (WT) cells, iAs treatment caused a dose-dependent reduction of the HIF1α protein (Fig. 4E), which further supports the hypothesis that iAs-induced HIF1α activation is Nrf2 dependent.

### Global mapping of Nrf2- and HIF1α-binding sites in the genome of the iAs-treated cells

**Figure 5:**
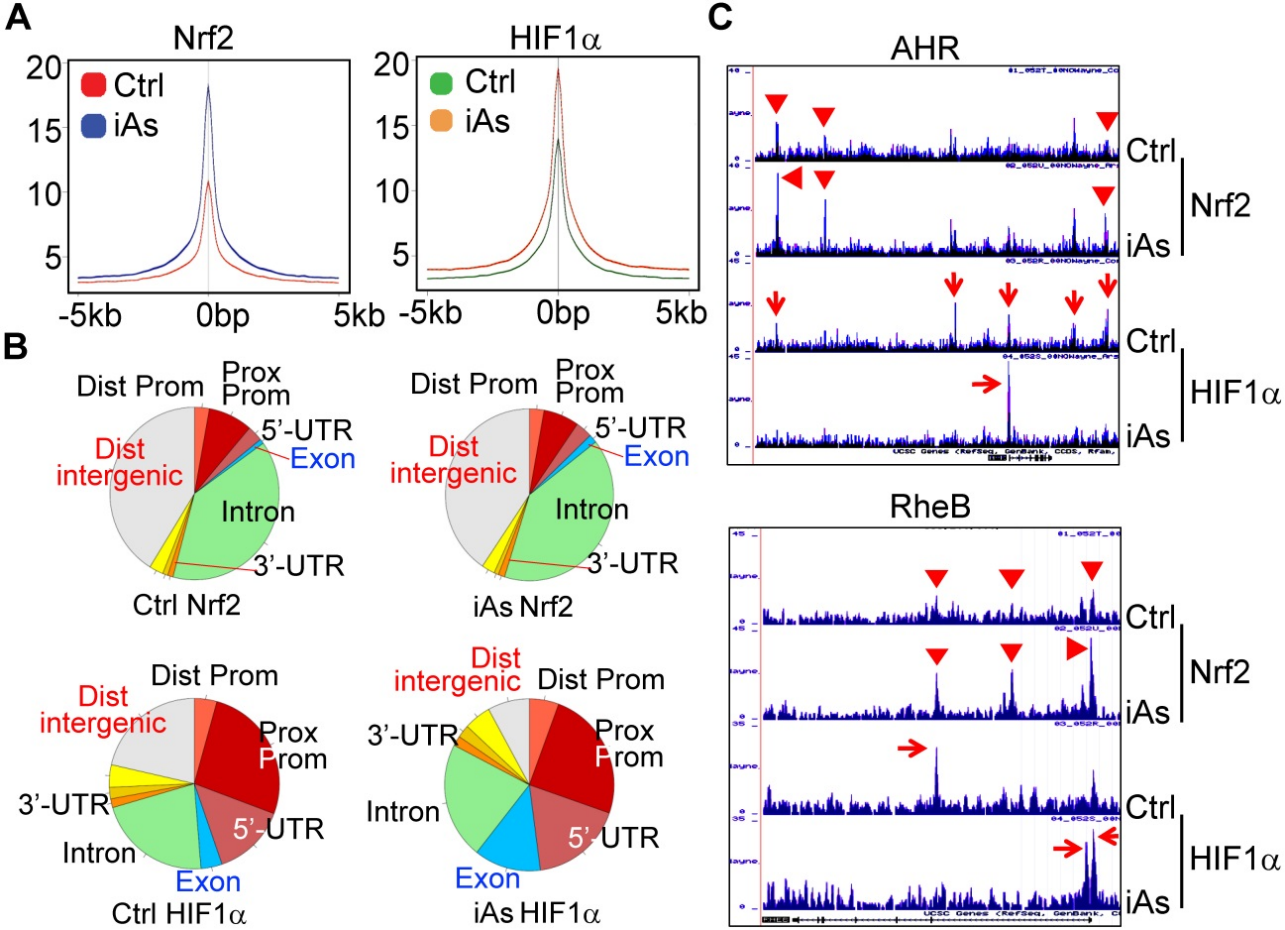
Global mapping of Nrf2- and HIF1α-binding in the control cells and the cells treated with 1 μM iAs for 6h. **A.** Trend plot showing average enrichment of control and iAs-induced Nrf2 (left) and HIF1α (right) signals over 5kb centered on the TSSs of gene promoters. **B.** Pie charts showing genome annotation of peaks in each cluster of Nrf2 (upper) and HIF1α (bottom) in the control cells (left) and iAs treated cells (right), respectively. **C.** Browser tracks showing enrichment of Nrf2 (red triangles) and HIF1α (red arrows) in genes of AHR (upper) and RheB (bottom) in response to iAs.

There are reports showing activation of either Nrf2 or HIF1α by iAs. However, these studies were mainly focused on the antioxidant response of Nrf2 and the angiogenesis effect of HIF1α, respectively. The metabolic regulation of both Nrf2 and HIF1α in response to iAs hasn't been studied. To better understand how Nrf2 and HIF1α contribute to the iAs-induced metabolic shift and the generation of the CSCs, we next performed chromatin immunoprecipitation coupled with deep sequencing (ChIP-seq) experiments to map the binding sites of Nrf2 and HIF1α in the control BEAS-2B cells and the cells treated with 1 μM iAs for 6h. The results of ChIP-seq revealed strong enrichments of Nrf2 and HIF1α in the promoter, gene body, and/or intergenic regions by iAs (Fig. 5A, merged peak regions). For the majority of genes, iAs treatment enhanced the enrichment of Nrf2, but did not cause shift of the enrichment peaks from one position to another on the individual gene locus (Fig. 5B), as exampled for AHR and RheB genes (Fig. 5C). In contrast, iAs not only amplified some of the pre-existing peaks of HIF1α on the genome, but shifted the location of HIF1α peaks on many genes also. Most significantly, iAs decreased HIF1α level in the distal intergenic region, but increased the occupancy of HIF1α in the exon and 5'-UTR regions of the genes (Fig. 5B). There are considerable HIF1α peaks in the intergenic regions as well as intron regions under the control condition, as indicated by red arrows on the gene loci of AHR and RheB (Fig. 5C). Upon iAs treatment, some of these intergenic peaks, such as the upstream and downstream HIF1α peaks of the AHR gene in the control cells, and the intronic HIF1α peak in the first intron of the RheB gene, disappeared, whereas the HIF1α peaks in the promoter and/or the first exon were enhanced substantially.

### iAs induces a concerted metabolic regulation by Nrf2 and HIF1α

**Figure 6:**
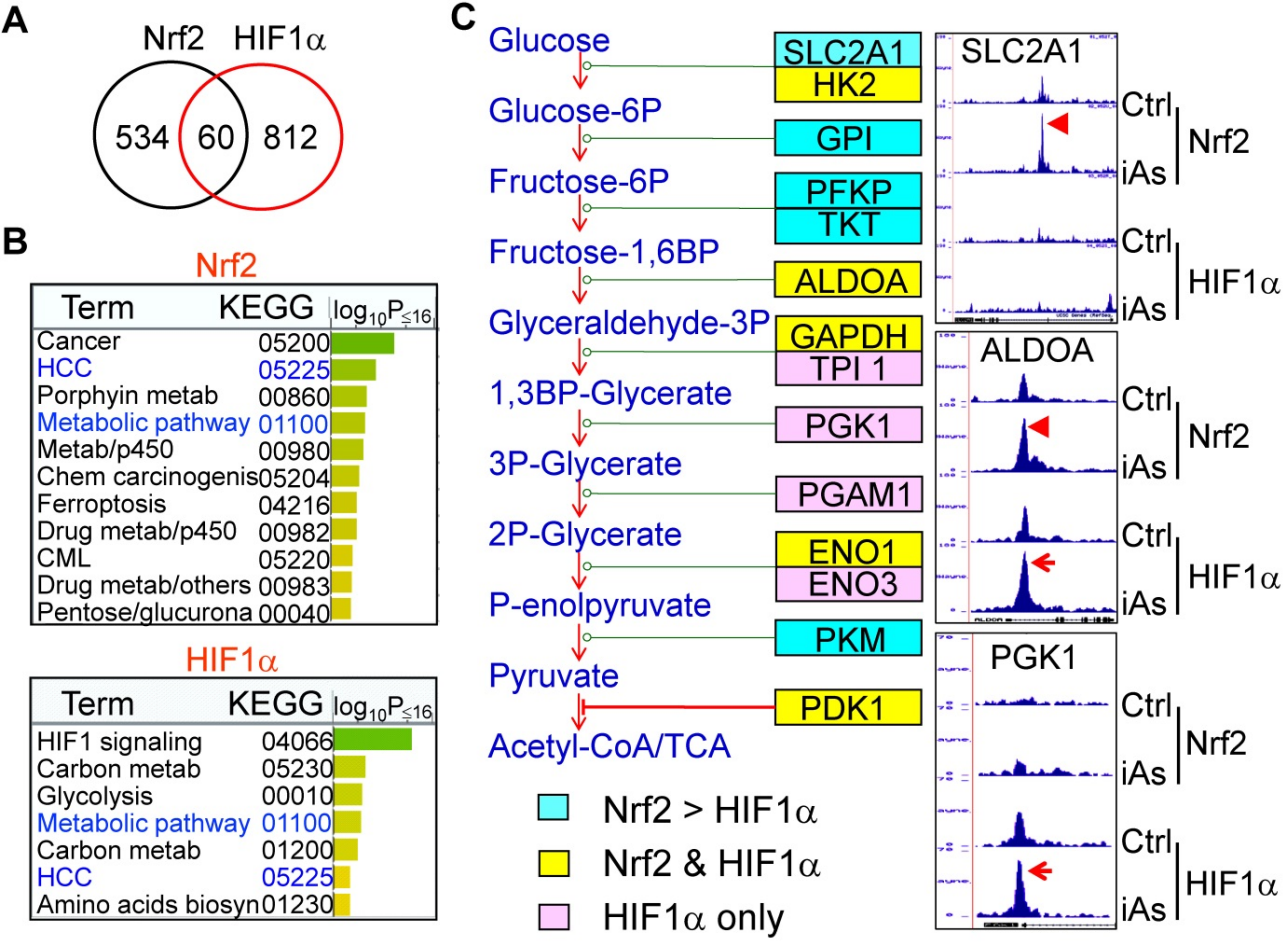
Concerted effects of iAs-induced Nrf2 and HIF1α on glycolytic metabolism. **A.** Venn diagrams showing overlapping of genes enriched with Nrf2 and HIF1α induced by iAs as determined by ChIP-seq. **B.** KEGG pathway analyses of the iAs-induced Nrf2-enriched genes (upper) and HIF1α-enriched genes (bottom). **C.** Diagram of glycolysis and the key enzymes at the indicated steps of glycolysis. Cyan color indicates the genes that are mainly enriched with Nrf2 in response to iAs; pink color indicates the genes that are mostly enriched with HIF1α induced by iAs; and yellow color shows the genes that are enriched with both Nrf2 and HIF1α in the cells treated with iAs. Right panels are the screenshot examples of the glycolytic genes enriched with Nrf2, HIF1α or both.

Global mapping by ChIP-seq showed that iAs induced a significantly enhanced binding of Nrf2 and HIF1α on 594 and 872 genes, respectively. Although only 60 genes showed overlap between Nrf2 and HIF1α in ChIP-seq (Fig. 6A), WikiPathway analysis (https://biit.cs.ut.ee/gprofiler/gost) revealed that both Nrf2 and HIF1α are the key regulators for the genes in metabolic and hepatocellular carcinoma (HCC) pathways (Fig. 6B). When ranked by significant levels, the top ranked pathways for the iAs-induced Nrf2-enriched genes are centered on the groups of genes in cancer, metabolism, p450 and drug metabolism, ferroptosis, and chemical carcinogenesis. As expected, the top pathways of iAs-induced HIF1α-enriched genes are those in HIF1 signaling, glycolysis, and metabolism. Indeed, as revealed by ChIP-seq, the key enzymes at each step of the central glycolytic pathway are enriched with either Nrf2 or HIF1α, or both (Fig. 6C). Nrf2 appears to be a dominant regulator for the enzymes of SLC2A1, GPI, PFKP, TKT, and PKM in cellular response to iAs. There is a cooperative regulation between Nrf2 and HIF1α for the genes of HK2, ALDOA, GAPDH, ENO1, and PDK1 in the iAs-treated cells. Other genes in this pathway, including TPI1, PGK1, PGAM1, and ENO3, are regulated mainly by HIF1α only in a manner of Nrf2 independent.

### Nrf2 and HIF1α mediate iAs-induced shunting of glycolysis into the HBP and serine/glycine pathway

**Figure 7:**
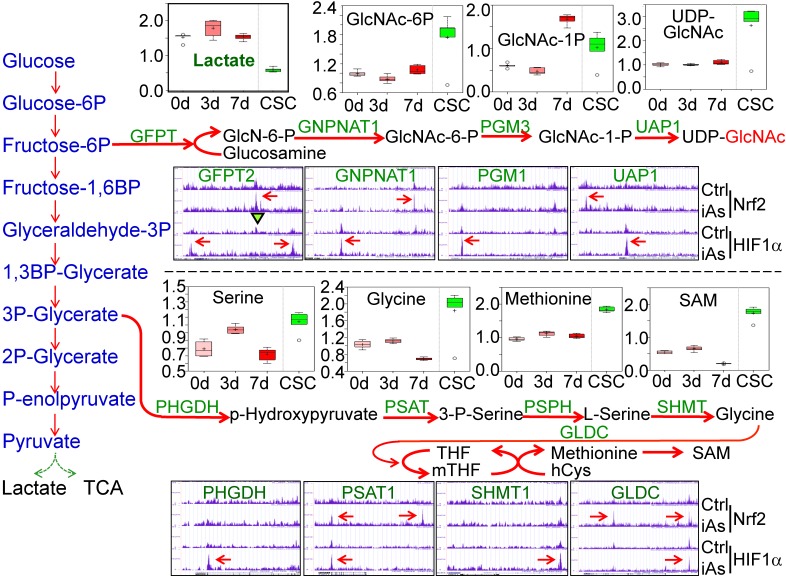
iAs-induced Nrf2 and HIF1α regulates the HBP and serine/glycine pathways of glycolysis. Upper half: Panels above the HBP pathway diagram show the actual levels of lactate and the major metabolites in HBP pathway as determined by quantitative metabolomics. Panels under the HBP show ChIP-seq data of the indicated HBP pathway genes. Lower half: Panels above the serine/glycine pathway diagram show the levels of the metabolites in this pathway as determined by quantitative metabolomics. Panels under the serine/glycine pathway diagram show ChIP-seq data of the key genes in this pathway. The iAs-induced enrichment of Nrf2 and/or HIF1α were indicated by red arrows.

The analysis of ^13^C-glucose flux indicated an active metabolism of glycolysis in the iAs-induced CSCs (Fig. 3). However, untargeted global metabolomics unraveled a substantial decrease of lactate in CSCs (Fig. 7, panel of Lactate), suggesting that the enhanced glycolysis in the iAs-induced CSCs may differ from the observed glycolytic pattern in the cancer cells and some normal stem cells in which glycolysis is preferentially linked to lactate production. Further analysis of the metabolomics data indicated that the majority of metabolic intermediators in the glycolytic pathway in CSCs are shunted to the side pathways of glycolysis, most notably, the hexosamine biosynthetic pathway (HBP) for protein O-GlcNAcylation (O-GlcNAc) and the serine-glycine pathway linked to one-carbon metabolism that is associated with the generation of S-adenosyl methionine (SAM) (Fig. 7.). In BEAS-2B cells, treatment of the cells with 0.25 μM iAs for 3 to 7 days induced notable increase of acetylglucosamine (GlcNAc), GlcNAc-6P and GlcNAc-1P. In the iAs-induced CSCs, the levels of these HBP pathway metabolites are significantly up-regulated. As indicated by ChIP-seq, the key genes in this pathway, including the rate-limiting enzyme, fructose-6-phosphate-amidotransferase (GFPT), GNPNAT1, PGM3, and UAP1, are enriched with either Nrf2, or HIF1α, or both, in response to iAs treatment (Fig. 7). Similarly, iAs also induced an enhanced enrichment of Nrf2 and HIF1α on PHGDH, PSAT, SHMT1, and GLCD that catalyze the serine-glycine pathway of the glycolysis, which is in agreement with the increased production of serine, glycine, methionine, and SAM as determined by metabolomics analysis in the CSCs (Fig. 7).

### iAs induces mutual regulation of Nrf2 and HIF1α

**Figure 8:**
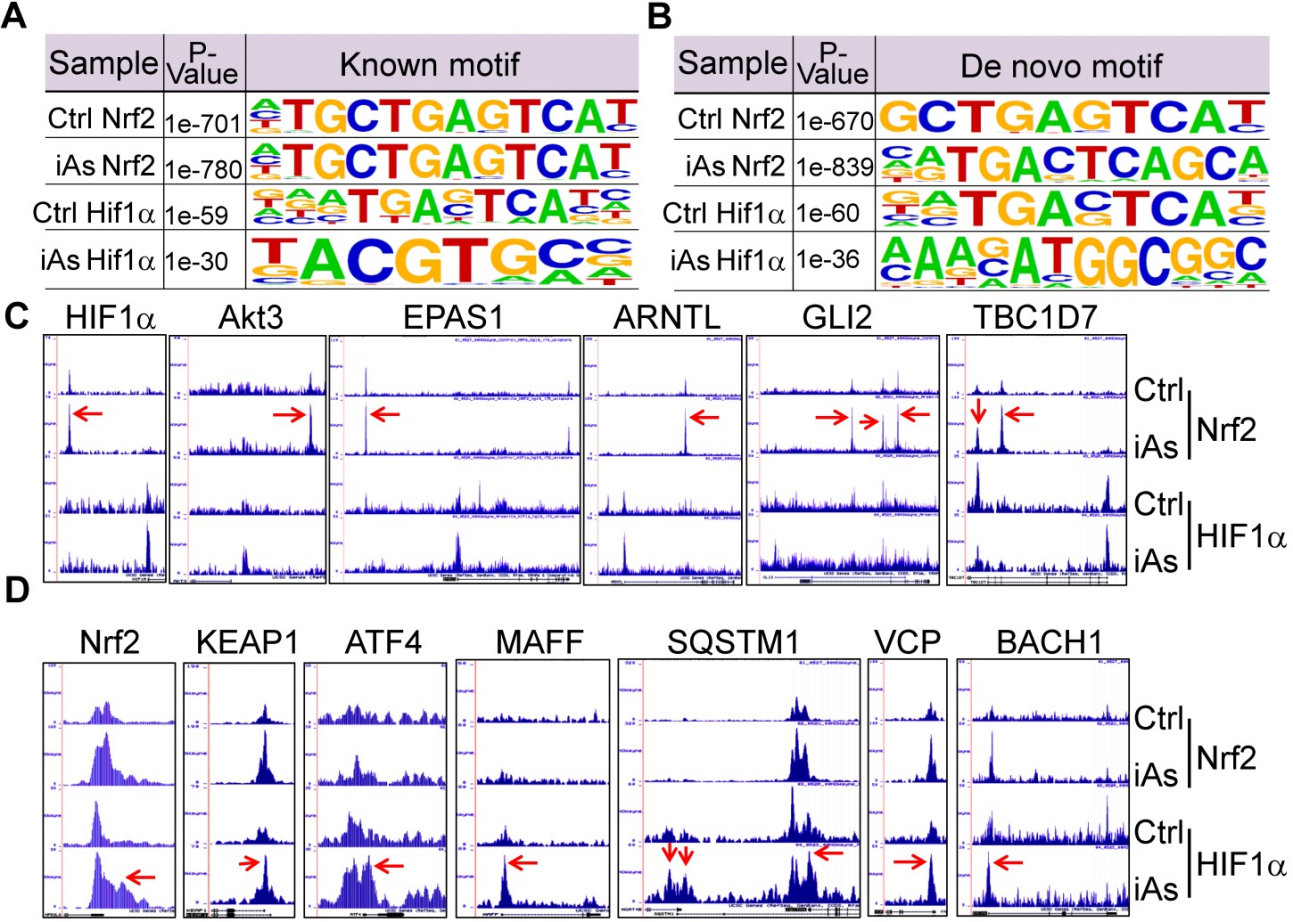
Mutual regulation of Nrf2 and HIF1α in response to iAs. **A.** Known Nrf2 and HIF1α motifs that are significantly enriched in the control cells and iAs-treated cells. P value was calculated in HOMER using a binomial test. **B.** *De novo* Nrf2 and HIF1α motifs that are significantly enriched in the control and iAs-treated cells. P values were calculated as in A. **C.** Screenshots of ChIP-seq Browser tracks of the HIF1α and the key HIF1α pathway genes that showed significant enrichment of Nrf2 induced by iAs as indicated by red arrows on the these gene loci. **D.** Screenshots of Browser tacks of the Nrf2 and the center Nrf2 pathway genes that showed significant enrichment of HIF1α (red arrows) induced by iAs on these indicated gene loci.

Using the findMotifs Genome program of the HOMER package, we performed known motif enrichment analysis by inputting sequences comprising +/-100 bp from the center of the top 1000 Nrf2 and HIF1α peaks, respectively. An identical known Nrf2 motif, TGCTGAGTCAT, as reported by Ney et al 21, was found between the control and iAs-treated cells (Fig.8A). The most enriched HIF1α motif in control cells contains **T**G/T**A**G/C/T**T**C/A**A**T/C/G core sequence, which roughly matches the 3'-terminal TGAGTCAT sequence of Nrf2 consensus binding motif and reflects the nature that HIF1α and Nrf2 peaks occurred at the same position under the control condition on many genes. In the iAs-treated cells, HIF1α motif is closely matched to the known HIF1α core motif, RCGTG 22, with a sequence preference of TACGTGCC. To address the possibility of alternate Nrf2 and HIF1α motifs, *de novo* motif analysis was performed also and indicated a slight variation of the iAs-induced Nrf2 binding sites, GCTGAGTCAT, from the Nrf2 binding sequence in control cells, caTGACTCAGCa, the same motif that had been reported by Malhotra et al 23 and Chorley et al 24 (Fig. 8B). Intriguingly, we found most of the Nrf2 motifs on the key glycolytic genes are the iAs-induced *de novo* Nrf2 motifs, such as motifs on SLC2A1, GPI, ENO1, PKM, PFKP, ETC. (sFig. 3 and data not shown). The *de novo* HIF1α motif in control cells is highly similar to the known HIF1α motif of the control cells, which appears to be similar to the *de novo* Nrf2 motif of the cells treated with iAs. A new HIF1α motif, AAAGATGGCGGC, was identified in the iAs-induced cells, which accounted for 14% of the total HIF1α peaks and has sequence similarity to the YY1 consensus sites (Fig. 8B). The known and *de novo* motif enrichment analyses, thus, suggested that at the control condition, Nrf2 and HIF1α might bind to the same sites for some genes. In response to iAs, HIF1α binds to new sites for gene regulation, which is in agreement with the findings that iAs treatment shifts HIF1α binding peaks from distal intergenic region to the exon and 5'-UTR regions (Figs. 5B and 5C).

As stated earlier, iAs-induced HIF1α accumulation is Nrf2-dependent (Fig. 4). By an additional manual search, we identified a strong Nrf2-enrichment peak in response to iAs at the 33kb upstream of the HIF1α gene (Fig. 8C). Motif search revealed a conserved known Nrf2 binding element, TGCTGAGTCAT, in this Nrf2-binding region (sFig. 4), which further confirmed Nrf2-dependent expression of HIF1α. In addition to HIF1α, Nrf2 appears to be able to induce transcription of several other genes that are involved in the transcription or the transcriptional activity of HIF1α in the cells treated with iAs, such as Akt3, EPAS1, ARNTL, GLI2, TBC1D7, Foxk1, etc. (Fig. 8C). Known or *de novo* Nrf2 motifs were found at the Nrf2 peaks in ChIP-seq in all of these genes. Rather than acting as a transcriptional partner, ARNTL is a direct transcription factor that binds to the HIF1α promoter to enhance HIF1α transcription 25. It is interesting to note that there is no Nrf2 enrichment peaks in response to iAs on those genes that encode the known negative regulators of HIF1α, including FIH1, VHL, HIF3α, EGLN1, EGLN2, Rbx1 etc., except EGLN3 that induces prolyl hydroxylation and the subsequent degradation of HIF1α (data not shown). Interestingly, ChIP-seq also indicated that iAs stimulated an enhanced HIF1α binding in the upstream, promoter, or gene body of the Nrf2 gene and the genes regulating Nrf2 activation or activity, including KEAP1, ATF4, MAFF, SQSTM1, VCP, BACH1 (Fig. 8D), and AHR (Fig. 5C), indicating a possible mutual positive regulation between Nrf2 and HIF1α. Intriguingly, an enrichment of Nrf2 was found in the first exon/intron of the gene encoding KEAP1, the negative regulator of Nrf2, in response to iAs. However, Westernblotting showed that iAs induced a dose dependent decrease of the KEAP1 protein (Fig. 4A), which could be prevented in the presence of proteasome inhibitor, MG132 (data not shown).

### Nrf2 and HIF1α orchestrate iAs-induced expression of the stemness genes for CSCs

**Figure 9:**
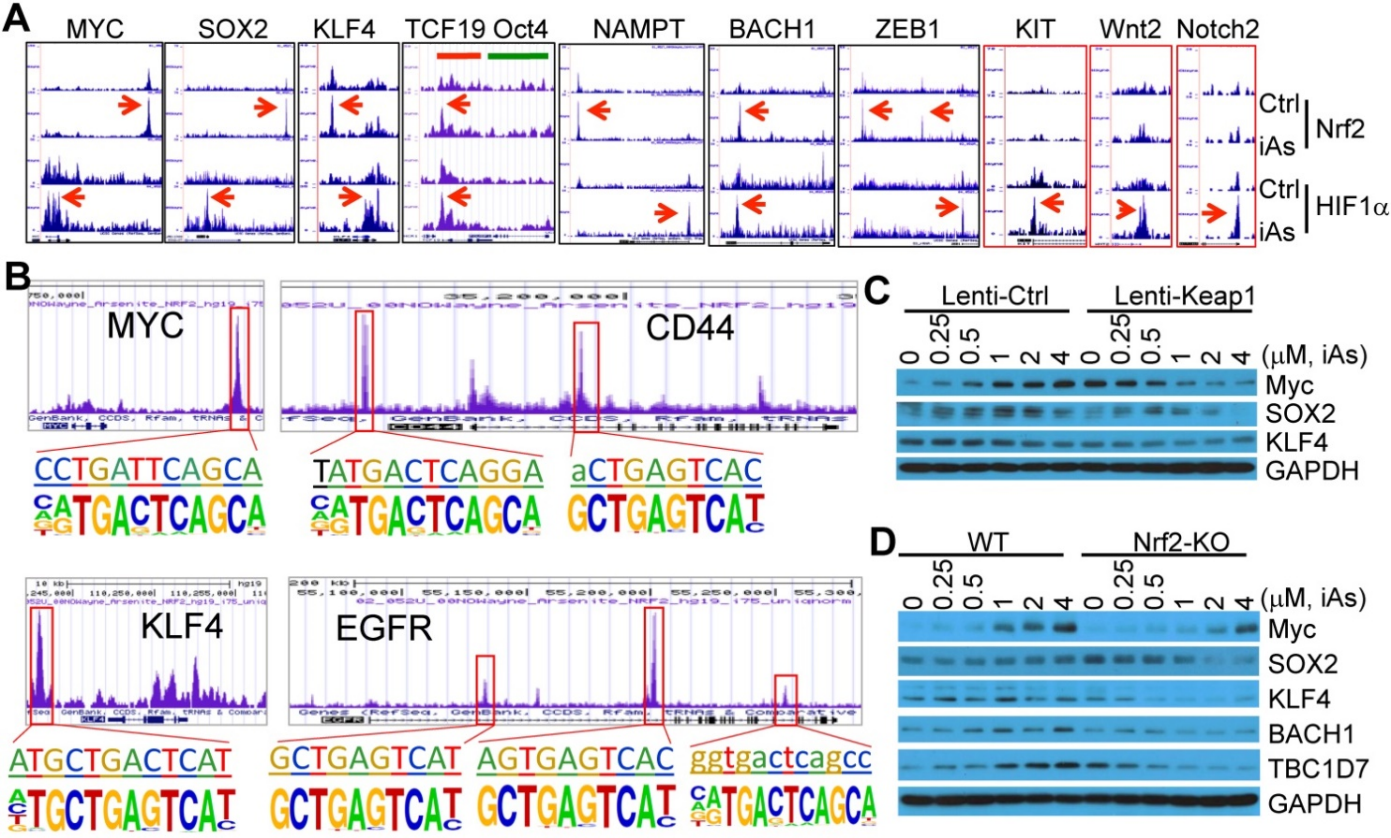
iAs induces expression of a number of stemness genes through Nrf2 and/or HIF1α activation. **A.** Enrichment status of Nrf2 and HIF1α (pointed by red arrows) induced by iAs on these indicated stemness genes as determined by ChIP-seq. **B.** *De novo* and/or known Nrf2 motif(s) induced by iAs on the promoter or gene body of MYC, CD44, KLF4, and EGFR that had been linked to the stemness of CSCs. **C.** Nrf2 inhibition by lentiviral Keap1 transfection prevented induction of MYC and SOX2 by iAs. This Nrf2 inhibition also reduced KLF4 expression. **D.** Nrf2 knockout blocked iAs-induced expression of MYC, SOX2, BACH1, TBC1D7, and decreased expression of KLF4.

Nrf2 has been viewed as a master regulator of the key hallmarks of cancer 8. Few studies explored possible contribution of Nrf2 signaling to the generation or function of the CSCs by focusing on a single gene important for the self-renewal or proliferation of the CSCs. It is unexpected that ChIP-seq data unraveled a number of stemness genes are regulated by Nrf2, HIF1α, or both (Fig. 9A). Myc, Sox2 and KLF4, three of the four Yamanake factors required for the pluripotency and self-renewal of the embryonic stem cells (ESC) and the induced pluripotent stem cells (iPSC), showed a strong enrichment of both Nrf2 and HIF1α in their gene loci in response to iAs. Similarly, NAMPT, ZEB1, BACH1, CD44, and EGFR, that are involved in EMT and CSCs 26-28, exhibited Nrf2 and HIF1α binding in the upstream and promoter regions, respectively (Figs. 9A and 9B). The two upstream Nrf2 peaks of ZEB1 have an identical iAs-induced *de novo* Nrf2 motif (sFig. 5). Both CD44 and EGFR have multiple Nrf2 peaks with *de novo* Nrf2 elements in promoter and gene body, respectively (Fig. 9B and sFig. 7). Although there is no notable binding of either Nrf2 or HIF1α on Oct4, another one of the four Yamanake factors, the immediate downstream gene, TCF19, showed both Nrf2 and HIF1α binding at the first and second exon regions. Motif search showed that most of these Nrf2 elements on these stemness genes match the *de novo* Nrf2 motifs in the iAs-treated cells and control cells, respectively, except the element at the upstream of KLF4 that represents the known Nrf2 motif in control or iAs-treated cells (Fig. 9B). In addition to the down-stream strong Nrf2 binding, the MYC gene also showed a significant enrichment of Nrf2 induced by iAs at the promoter, the first and second exons, although neither known nor *de novo* Nrf2 motif was identified at these regions (Figs. 9A and 9B). Several other stemness genes, including KIT, Wnt2 and Notch2, showed HIF1α-binding only that was enhanced by iAs.

To verify the importance of Nrf2 on the expression of these stemness genes, we determined the protein levels of the representative stemness genes in the cells where Nrf2 was inhibited by lentiviral-based Keap1 overexpression or genetically disrupted by CRISPR-Cas9 gene editing (Figs. 4D and 4E). As depicted in Fig. 9C, iAs induced Myc and SOX2 in a dose dependent manner in the cells expressing a control lentiviral vector. This induction was prevented by the expression of exogenous Keap1. The level of KLF4 appears to be not affected by iAs treatment, neither in control vector nor in Keap1 expressing cells. However, inhibition of Nrf2 by overexpression of Keap1 substantially reduced the level of KLF4. Similarly, knockout of Nrf2 reduced or blocked induction of Myc and SOX2 by iAs (Fig. 9D). A pronounced decrease of KLF4 was observed in the Nrf2 KO cells relative to the wild-type (WT) cells. In addition to these well-established stemness genes, we also found that iAs induced a Nrf2 dependent expression of BACH1, a most recently identified stemness transcription factor in human embryonic stem cells (hESCs) (Fig. 9D and sFig. 6) 29, and TBC1D7 that boosts Akt activation through binding to the negative regulator of Akt, PHLPP1 30 (Fig. 9D and sFig. 6). It should be noted that TBC1D8, another member of the TBC domain GTPase activating protein, had recently been shown to be able to drive metabolic reprogramming in cancer cells 31. Thus, in agreement with the data of ChIP-seq, these results demonstrated that in addition to the classical antioxidant genes and major metabolic genes, Nrf2 either alone or in combination with HIF1α, is a direct transcription factor for the iAs-induced expression of the central stemness genes critical for the self-renewal and multipotency of the CSCs.

## Discussion

The carcinogenic role of environmental iAs exposure had been well-recognized in the past decades. The molecular mechanism of how iAs contributes to cancer development, however, is still not fully understood. Some studies indicated that iAs can either convert normal stem cells to CSCs or induce generation of CSCs from the well differentiated cells 4, 6, 7. Meanwhile, earlier studies had demonstrated an epithelial to mesenchymal transition (EMT) morphology, a feature shared by many types of CSCs, of the iAs-transformed endothelial cells and liver epithelial cells 32, 33. Despite possible heterogeneity of CSCs for a given tumor, all CSCs possess two of the most important characteristics: the ability to self-renew and the capacity to repopulate the phenotypically heterogeneous cancer cells, which are believed to be the sources of chemodrug resistance, tumor recurrence and metastasis. Meanwhile, emerging evidence indicates that metabolic reprogramming, especially. the shift of glucose metabolism from mitochondrial TCA cycle to aerobic glycolysis, the Warburg effect, is a pre-required step for the generation of the CSCs or the iPSCs 34. In the iAs-induced CSCs, both untargeted global metabolomics and ^13^C-glucose tracing indicated that the majority of the glycolytic metabolic intermediates are shunted into the HBP for protein O-GlcNAcylation (O-GlcNAc) and the serine-glycine pathway linked to one-carbon metabolism that is associated with the generation of S-adenosyl methionine (SAM). This metabolic feature of the iAs-induced CSCs may differ from the lactate generation of glycolysis for some cancer cells and normal stem cells.

Mechanistically, our data suggested that Nrf2 may be the one of the key factors in mediating iAs-induced generation of the CSCs. This effect of Nrf2 is very likely achieved through two mutually cooperative pathways regulated by Nrf2. First, iAs induces a Nrf2-dependent HIF1α activation, which in turn coordinate with Nrf2 for the transcription of genes in glycolysis and the side pathways of glycolysis, such as HBP and serine-glycine pathway. Secondly, both Nrf2 and HIF1α are direct transcription factors for the expression of a wide spectrum of genes critical for the acquisition of cellular stemness, including MYC, SOX2, KLF4, NAMPT, KIT, BACH1, ZEB1, etc. Few previous reports using the bioinformatics or biochemical approaches had identified a Nrf2 binding motif at the upstream of HIF1α locus 35, 36. The ChIP-seq results presented in this report clearly indicate that this Nrf2 binding is highly inducible by iAs. Inhibition or genetic deletion of Nrf2 prevented HIF1α induction by iAs, which further substantiated the conclusion of Nrf2 dependency of HIF1α activation by iAs. During iPSC reprogramming of human fibroblasts, it is believe that Nrf2 dependent HIF1α expression is essential for the metabolic shift from OXPHOS to glycolysis 36. Indeed, the role of HIF1α in promoting glycolytic gene expression in adult stem cells and CSCs had long been recognized 37-40. In the case of iAs-induced CSCs, however, the establishment of glycolytic metabolism might be multifactorial. Nrf2 itself appears to be a master regulator for several rate limiting enzymes in both the main glycolytic pathway and the side pathways of glycolysis, which certainly be further re-enforced by the iAs-induced, Nrf2 dependent HIF1α expression.

It was estimated that approximately 30% of human lung cancers acquire mutations of the Nrf2 signaling genes, including Keap1 and Nfe2l2 that encodes Nrf2 protein, which leads to a stabilization and sustained activation of Nrf2 27. This notion was supported by the fact that many types of human cancers showed an elevated activation of Nrf2 8, 10. Whether Nrf2 contributes to the maintenance and function of the stem cells and CSCs, however, remains controversial. In haematopoietic stem cells (HSCs), Nrf2 represses expansion of HSC and progenitor cell compartment, possibly through upregulating CXCR4 41. In hESCs, however, high Nrf2 activity may contribute to the self-renewal and pluripotency 42. More importantly, Nrf2 silencing by shRNA reduced tumor sphere formation of the glioblastoma stem cells (GSC) 43 and enhanced differentiation of the ALDH1A1 positive ovarian CSCs 11. In ChIP-seq of the iAs-treated cells, our data clearly indicated a direct regulation of Nrf2 on a number of key stemness genes. Mounting evidence had established fundamental roles of the central stemness factors, including MYC, SOX2, KLF4, KIT, Wnt, Notch, etc, in hESCs, iPSCs, adult stem cells, and CSCs. It is worth noting that in addition to these traditional stemness factors, Nrf2 as well as HIF1α is also actively involved in the regulation of several newly identified genes crucial for the self-renewal and pluripotency/multipotency of the stem cells and CSCs. NAMPT, a rate-limiting enzyme in a major NAD^+^ biosynthetic pathway, had recently been shown to be able to induce colon and glioblastoma CSCs through pathways that control stemness and EMT 28, 44. BACH1, a previously reported BTB and CNC homology transcription factor that competes with Nrf2 for MAF binding, had recently been shown to be able to maintain self-renewal and impedes differentiation of the hESCs through recruiting deubiquitinase Usp7 and stabilizing Nanog, SOX2 and OCT4 29. Most recently, studies by Lignitto et al indicated that Nrf2 induces metastasis of the lung adenocarcinoma in a BACH1-dependent manner 27. Sustained Nrf2 activation upregulates home oxygenase-1 (Ho-1) that catabolizes free heme, causing functional inactivation of ubiquitin ligase Fbxo22 and the subsequent stabilization of BACH1 and tumor cell metastasis. The identification of a known Nrf2 motif in the first exon/intron region of the BACH1 gene (Fig. 9A and sFig. 6) indicates that in addition to the heme catabolism-associated protein stabilization, direct transcriptional regulation of Nrf2 on BACH1 is another layer of Nrf2-BACH1 interaction. This notion is further strengthened by the prevention of iAs-induced accumulation of BACH1 protein in the cells were Nrf2 gene was disrupted through CRISPR-Cas9 gene editing. ZEB1, a zinc finger E-box transcription factor, has long been viewed as one of the master regulators of epithelial-to-mesenchymal transition (EMT), a characteristic possessed by most of the CSCs 26. In human basal breast cancers, ZEB1 is instrumental in mediating the dedifferentiation of the non-CSCs to CSC state 45. It was found that some non-CSCs are plastic, and the promoter of ZEB1 gene in these cells is in a bivalent chromatin configuration, enabling it to be rapidly transcribed in response to microenvironmental signals. It is very likely, thus, the enhanced enrichment of Nrf2 at the two major Nrf2 motifs in the upstream of ZEB1 gene induced by iAs may promote the conversion of ZEB1 promoter from the bivalent to active chromatin configuration. The pre-metastatic role of BACH1 and pre-EMT of ZEB1 further strengthens the hypothesis that Nrf2 promotes iAs-induced generation of CSCs, since extensive evidence confirmed that CSCs are intrinsically more prone to disseminate and possess properties of EMT.

Overwhelming evidence suggested rapid degradation of HIF1α under normoxia due to the PHD-dependent prolyl hydroxylation and VHL-mediated ubiquitination of the HIF1α protein 46. Intriguingly, HIF1α signaling was selectively activated in CSCs of mouse lymphoma and human acute myeloid leukemia (AML) under normoxia 47. It was believed that normoxia HIF1α activation is essential for the maintenance of CSCs population through enhancing the expression of the Notch-targeting gene, Hes1. Indeed, both Nrf2 and HIF1α signals was significantly enriched as demonstrated in ChIP-seq in the upstream, promoter and gene body of the Hes1 gene in the iAs-treated cells (sFig. 6). Although limited information is available on how HIF1α was activated under normoxia, it is very likely that Nrf2-dependent transcription of HIF1α, and the subsequent mutual regulation between Nrf2 and HIF1α signaling pathways as indicated in this report, may be the key for normoxia HIF1α activation and the acquisition of the CSC features in response to iAs. Since Nrf2 had been viewed as one of the key factors in development and developmental toxicology 48, it is also important to consider the Nrf2-dependent HIF1α activation as a pivot measure in maintaining the normal stem cells and embryonic development. An intricate interaction or cross-talk between Nrf2 and aryl hydrocarbon receptor (AHR) had been reported before 49, 50. The Nrf2 element on the upstream of HIF1α and other genes has some similarities with the sequence of the AHR binding motif. Thus, it is possible that both Nrf2 and AHR participated in the transcription of HIF1α. Interestingly, AHR had been shown to cooperate with HIF1α through the commonly shared partner, AHR nuclear translocator (ARNT/HIF1β), to orchestrate glycolysis and tumor microenvironment 51. The finding that iAs induced a strong enrichment of both Nrf2 and HIF1α on the AHR gene (Fig. 5C) further solidified the role of cross-talk among Nrf2, HIF1α and AHR in the metabolic reprogramming and the establishment of the CSCs features.

From a therapeutic standpoint, the current report also opens the door to explore whether targeting Nrf2 can improve therapeutic outcome for cancer patients by specifically blocking the Nrf2 activity on HIF1α, glycolysis and the stemness genes to eliminate CSCs. Although transcription factors are generally viewed as poor drug targets, various compounds had been developed to inhibit the activation of Nrf2, and some of which had shown promising specificity. Moreover, in the iAs-induced malignant transformation and the generation of CSCs, the data presented in this report clearly indicated a critical role of Nrf2 played during these processes. Thus, the availability of various inhibitors suggests that it is possible to disrupt Nrf2 as well as HIF1α pathway for preventive gain of the human cancers resulted from environmental or occupational iAs exposure. Nevertheless, the development of therapies and preventions based on the activated Nrf2 and/or HIF1α signaling requires a more comprehensive understanding on how these transcription factors govern cell fate decisions and the interweaving pathways of Nrf2-dependent and Nrf2-independent HIF1α activation.

## Supplementary Material

Supplementary figures.

## References

[1] Chen QY, Costa M (2018). PI3K/Akt/mTOR Signaling Pathway and the Biphasic Effect of Arsenic in Carcinogenesis. Mol Pharmacol.

[2] Li L, Bi Z, Wadgaonkar P, Lu Y, Zhang Q, Fu Y (2019). Metabolic and epigenetic reprogramming in the arsenic-induced cancer stem cells.

[3] Upadhyay MK, Shukla A, Yadav P, Srivastava S (2019). A review of arsenic in crops, vegetables, animals and food products. Food Chem.

[4] Chang Q, Chen B, Thakur C, Lu Y, Chen F (2014). Arsenic-induced sub-lethal stress reprograms human bronchial epithelial cells to CD61 cancer stem cells. Oncotarget.

[5] Bain LJ, Liu JT, League RE (2016). Arsenic inhibits stem cell differentiation by altering the interplay between the Wnt3a and Notch signaling pathways. Toxicol Rep.

[6] Tokar EJ, Qu W, Liu J, Liu W, Webber MM, Phang JM (2010). Arsenic-specific stem cell selection during malignant transformation. J Natl Cancer Inst.

[7] Xu Y, Tokar EJ, Sun Y, Waalkes MP (2012). Arsenic-transformed malignant prostate epithelia can convert noncontiguous normal stem cells into an oncogenic phenotype. Environ Health Perspect.

[8] Rojo de la Vega M, Chapman E, Zhang DD (2018). NRF2 and the Hallmarks of Cancer. Cancer Cell.

[9] Ni HM, Woolbright BL, Williams J, Copple B, Cui W, Luyendyk JP (2014). Nrf2 promotes the development of fibrosis and tumorigenesis in mice with defective hepatic autophagy. J Hepatol.

[10] Feng L, Li J, Yang L, Zhu L, Huang X, Zhang S (2017). Tamoxifen activates Nrf2-dependent SQSTM1 transcription to promote endometrial hyperplasia. Theranostics.

[11] Kim D, Choi BH, Ryoo IG, Kwak MK (2018). High NRF2 level mediates cancer stem cell-like properties of aldehyde dehydrogenase (ALDH)-high ovarian cancer cells: inhibitory role of all-trans retinoic acid in ALDH/NRF2 signaling. Cell Death Dis.

[12] Wakabayashi N, Shin S, Slocum SL, Agoston ES, Wakabayashi J, Kwak MK (2010). Regulation of notch1 signaling by nrf2: implications for tissue regeneration. Sci Signal.

[13] Mathieu J, Zhang Z, Zhou W, Wang AJ, Heddleston JM, Pinna CM (2011). HIF induces human embryonic stem cell markers in cancer cells. Cancer Res.

[14] Aono J, Yanagawa T, Itoh K, Li B, Yoshida H, Kumagai Y (2003). Activation of Nrf2 and accumulation of ubiquitinated A170 by arsenic in osteoblasts. Biochem Biophys Res Commun.

[15] Person RJ, Ngalame NN, Makia NL, Bell MW, Waalkes MP, Tokar EJ (2015). Chronic inorganic arsenic exposure in vitro induces a cancer cell phenotype in human peripheral lung epithelial cells. Toxicol Appl Pharmacol.

[16] Q (2020). Z, Thakur C, Fu Y, Bi Z, Wadgaonkar P, Xu P, et al. Mdig promotes oncogenic gene expression through antagonizing repressive histone methylation markers. Theranostics.

[17] Scarpulla RC (2002). Nuclear activators and coactivators in mammalian mitochondrial biogenesis. Biochim Biophys Acta.

[18] Cullinan SB, Zhang D, Hannink M, Arvisais E, Kaufman RJ, Diehl JA (2003). Nrf2 is a direct PERK substrate and effector of PERK-dependent cell survival. Mol Cell Biol.

[19] Sun Z, Huang Z, Zhang DD (2009). Phosphorylation of Nrf2 at multiple sites by MAP kinases has a limited contribution in modulating the Nrf2-dependent antioxidant response. PLoS One.

[20] Tao S, Liu P, Luo G, Rojo de la Vega M, Chen H, Wu T (2017). p97 Negatively Regulates NRF2 by Extracting Ubiquitylated NRF2 from the KEAP1-CUL3 E3 Complex.

[21] Ney PA, Sorrentino BP, Lowrey CH, Nienhuis AW (1990). Inducibility of the HS II enhancer depends on binding of an erythroid specific nuclear protein. Nucleic Acids Res.

[22] Semenza GL, Jiang BH, Leung SW, Passantino R, Concordet JP, Maire P (1996). Hypoxia response elements in the aldolase A, enolase 1, and lactate dehydrogenase A gene promoters contain essential binding sites for hypoxia-inducible factor 1. J Biol Chem.

[23] Malhotra D, Portales-Casamar E, Singh A, Srivastava S, Arenillas D, Happel C (2010). Global mapping of binding sites for Nrf2 identifies novel targets in cell survival response through ChIP-Seq profiling and network analysis. Nucleic Acids Res.

[24] Chorley BN, Campbell MR, Wang X, Karaca M, Sambandan D, Bangura F (2012). Identification of novel NRF2-regulated genes by ChIP-Seq: influence on retinoid X receptor alpha. Nucleic Acids Res.

[25] Wu Y, Tang D, Liu N, Xiong W, Huang H, Li Y (2017). Reciprocal Regulation between the Circadian Clock and Hypoxia Signaling at the Genome Level in Mammals. Cell Metab.

[26] Caramel J, Ligier M, Puisieux A (2018). Pleiotropic Roles for ZEB1 in Cancer. Cancer Res.

[27] Lignitto L, LeBoeuf SE, Homer H, Jiang S, Askenazi M, Karakousi TR (2019). Nrf2 Activation Promotes Lung Cancer Metastasis by Inhibiting the Degradation of Bach1. Cell.

[28] Lucena-Cacace A, Otero-Albiol D, Jimenez-Garcia MP, Munoz-Galvan S, Carnero A (2018). NAMPT Is a Potent Oncogene in Colon Cancer Progression that Modulates Cancer Stem Cell Properties and Resistance to Therapy through Sirt1 and PARP. Clin Cancer Res.

[29] Wei X, Guo J, Li Q, Jia Q, Jing Q, Li Y (2019). Bach1 regulates self-renewal and impedes mesendodermal differentiation of human embryonic stem cells. Sci Adv.

[30] Madigan JP, Hou F, Ye L, Hu J, Dong A, Tempel W (2018). The tuberous sclerosis complex subunit TBC1D7 is stabilized by Akt phosphorylation-mediated 14-3-3 binding. J Biol Chem.

[31] Chen M, Sheng XJ, Qin YY, Zhu S, Wu QX, Jia L (2019). TBC1D8 Amplification Drives Tumorigenesis through Metabolism Reprogramming in Ovarian Cancer. Theranostics.

[32] Wang Z, Humphries B, Xiao H, Jiang Y, Yang C (2013). Epithelial to mesenchymal transition in arsenic-transformed cells promotes angiogenesis through activating beta-catenin-vascular endothelial growth factor pathway. Toxicol Appl Pharmacol.

[33] Chen C, Yang Q, Wang D, Luo F, Liu X, Xue J (2018). MicroRNA-191, regulated by HIF-2alpha, is involved in EMT and acquisition of a stem cell-like phenotype in arsenite-transformed human liver epithelial cells. Toxicol In Vitro.

[34] Thakur C, Chen F (2019). Connections between metabolism and epigenetics in cancers.

[35] Lacher SE, Levings DC, Freeman S, Slattery M (2018). Identification of a functional antioxidant response element at the HIF1A locus. Redox Biol.

[36] Hawkins KE, Joy S, Delhove JM, Kotiadis VN, Fernandez E, Fitzpatrick LM (2016). NRF2 Orchestrates the Metabolic Shift during Induced Pluripotent Stem Cell Reprogramming. Cell Rep.

[37] Palomaki S, Pietila M, Laitinen S, Pesala J, Sormunen R, Lehenkari P (2013). HIF-1alpha is upregulated in human mesenchymal stem cells. Stem Cells.

[38] Finley LW, Carracedo A, Lee J, Souza A, Egia A, Zhang J (2011). SIRT3 opposes reprogramming of cancer cell metabolism through HIF1alpha destabilization. Cancer Cell.

[39] Peng G, Liu Y (2015). Hypoxia-inducible factors in cancer stem cells and inflammation. Trends Pharmacol Sci.

[40] Boso D, Rampazzo E, Zanon C, Bresolin S, Maule F, Porcu E (2019). HIF-1alpha/Wnt signaling-dependent control of gene transcription regulates neuronal differentiation of glioblastoma stem cells. Theranostics.

[41] Tsai JJ, Dudakov JA, Takahashi K, Shieh JH, Velardi E, Holland AM (2013). Nrf2 regulates haematopoietic stem cell function. Nat Cell Biol.

[42] Jang J, Wang Y, Kim HS, Lalli MA, Kosik KS (2014). Nrf2, a regulator of the proteasome, controls self-renewal and pluripotency in human embryonic stem cells. Stem Cells.

[43] Zhu J, Wang H, Sun Q, Ji X, Zhu L, Cong Z (2013). Nrf2 is required to maintain the self-renewal of glioma stem cells. BMC Cancer.

[44] Lucena-Cacace A, Umeda M, Navas LE, Carnero A (2019). NAMPT as a Dedifferentiation-Inducer Gene: NAD(+) as Core Axis for Glioma Cancer Stem-Like Cells Maintenance. Front Oncol.

[45] Chaffer CL, Marjanovic ND, Lee T, Bell G, Kleer CG, Reinhardt F (2013). Poised chromatin at the ZEB1 promoter enables breast cancer cell plasticity and enhances tumorigenicity. Cell.

[46] Ivan M, Kaelin WG Jr (2017). The EGLN-HIF O2-Sensing System: Multiple Inputs and Feedbacks. Mol Cell.

[47] Wang Y, Liu Y, Malek SN, Zheng P, Liu Y (2011). Targeting HIF1alpha eliminates cancer stem cells in hematological malignancies. Cell Stem Cell.

[48] Hahn ME, Timme-Laragy AR, Karchner SI, Stegeman JJ (2015). Nrf2 and Nrf2-related proteins in development and developmental toxicity: Insights from studies in zebrafish (Danio rerio). Free Radic Biol Med.

[49] Rousseau ME, Sant KE, Borden LR, Franks DG, Hahn ME, Timme-Laragy AR (2015). Regulation of Ahr signaling by Nrf2 during development: Effects of Nrf2a deficiency on PCB126 embryotoxicity in zebrafish (Danio rerio). Aquat Toxicol.

[50] Hahn ME, McArthur AG, Karchner SI, Franks DG, Jenny MJ, Timme-Laragy AR (2014). The transcriptional response to oxidative stress during vertebrate development: effects of tert-butylhydroquinone and 2,3,7,8- tetrachlorodibenzo-p-dioxin. PLoS One.

[51] Gabriely G, Wheeler MA, Takenaka MC, Quintana FJ (2017). Role of AHR and HIF-1alpha in Glioblastoma Metabolism. Trends Endocrinol Metab.

