# Nrf2 and HIF1α converge to arsenic-induced metabolic reprogramming and the formation of the cancer stem-like cells: Erratum

**DOI:** 10.7150/thno.108244

**Published:** 2025-03-15

**Authors:** Zhuoyue Bi, Qian Zhang, Yao Fu, Priya Wadgaonkar, Wenxuan Zhang, Bandar Almutairy, Liping Xu, M'Kya Rice, Yiran Qiu, Chitra Thakur, Fei Chen

**Affiliations:** 1 Department of Pharmaceutical Sciences, Eugene Applebaum College of Pharmacy and Health Sciences, Wayne State University, 259 Mack Avenue, Detroit, MI 48201, USA; 2 School of Health Sciences, Wuhan University, 115 Donghu Road, Wuhan 430071, China; 3 Hubei Provincial Key Laboratory of Applied Toxicology, Hubei Provincial Center for Disease Control and Prevention, 8 Zhudaoquanbei Road, Wuhan 430079, China; 4 College of Pharmacy, Al-Dawadmi Campus, Shaqra University, P.O.Box 11961, Riyadh, Kingdom of Saudi Arabia.

In the originally published version of this article, panels in Figure 1 were inadvertently replaced with previously published images from *Oncotarget* (5:1290, 2014, Fig. 4E) and *Seminars in Cancer Biology* (57:10, 2019, Fig. 1A & 1B). These errors occurred during the process of referencing previously assembled images from our prior publications and compiling figures for the new manuscript. We have retrieved the original data and provided the corrected figure panels below. These corrections do not impact the interpretation of the data or the conclusions of the manuscript. We extend our gratitude to the anonymous reader who identified the error in Fig. 1A. The authors sincerely regret any confusion these errors may have caused.

## Figures and Tables

**Figure 1:**
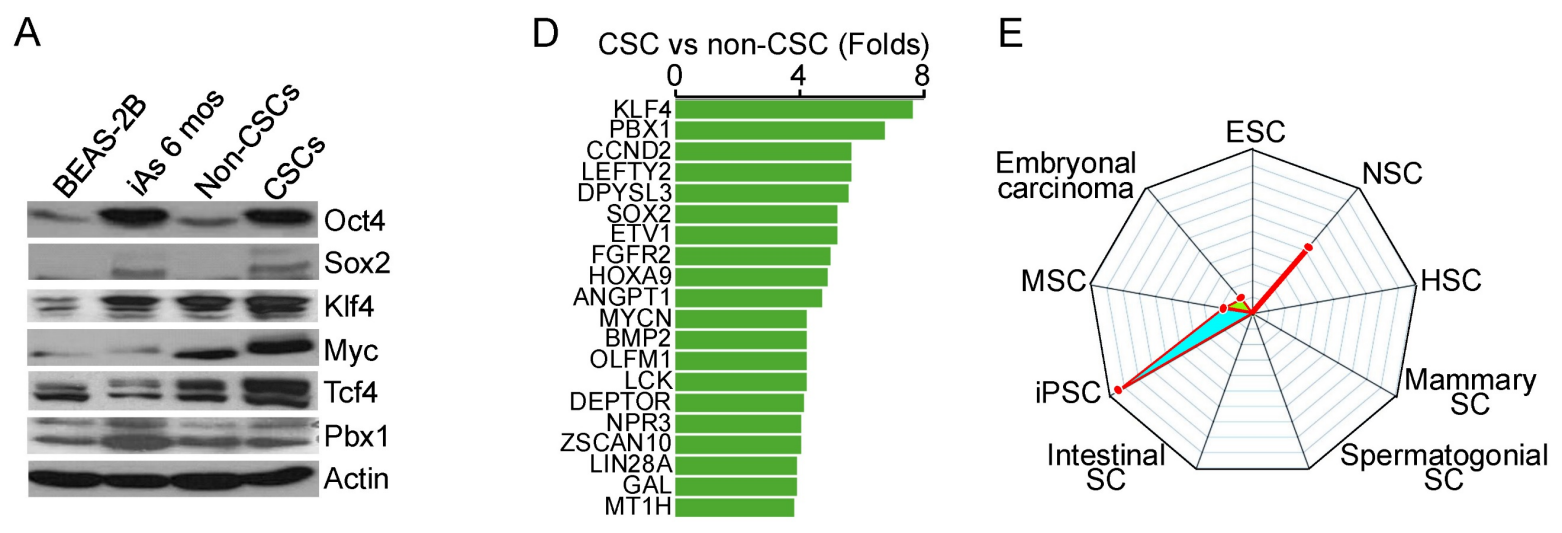
Correct images.

