# Supplementary material for: Nrf2 and HIF1α converge to arsenic-induced metabolic reprogramming and the formation of the cancer stem-like cells
Source: Theranostics. 2020 Mar 4;10(9):4134–49. doi: 10.7150/thno.42903 (PMC7086359; doi:10.7150/thno.42903)
Supplement: Supplementary file 1 — Supplementary figures. [file thnov10p4134s1.pdf]

Supplement Figure legends

sFig. 1. A. WikiPathway of the down-regulated genes in the iAs-induced CSCs. B. Down-regulation of genes for the respiratory chain of mitochondria in the iAs-induced CSCs. The minus numbers indicate fold decrease relative to the non-CSCs of these individual genes.

sFig. 2. Screening of Nrf2 knockout (KO) cells from the BEAS-2B cells subjected to CRISPR-Cas9 gene editing using sgRNA1 and sgRNA2 as indicated, respectively. The successful Nrf2 knockout rates are 15% for sgRNA1 and 60% for sgRNA2, respectively.

sFig. 3. Nrf2 element(s) on the glycolytic genes as exampled for ENO1, GPI and GAPDH.

sFig. 4. Identification of a conserved known Nrf2 element at the 32.5 kb upstream of the transcription stat site of HIF1α gene.

sFig. 5. Multiple Nrf2 elements identified at the upstream of the ZEB1 gene.

sFig. 6. Known and de novo Nrf2 binding motifs in the gene loci of BACH1, Hes1 and TBC1D7. Red arrows indicate the iAs-induced HIF1α binding.

sFig. 7. Cooperative regulation of Nrf2 and HIF1α on NAMPT, CD44 and EGFR. Known and/or de novo Nrf2 motifs were indicated. Red arrows indicate the iAs-induced HIF1α binding.

sFig.1

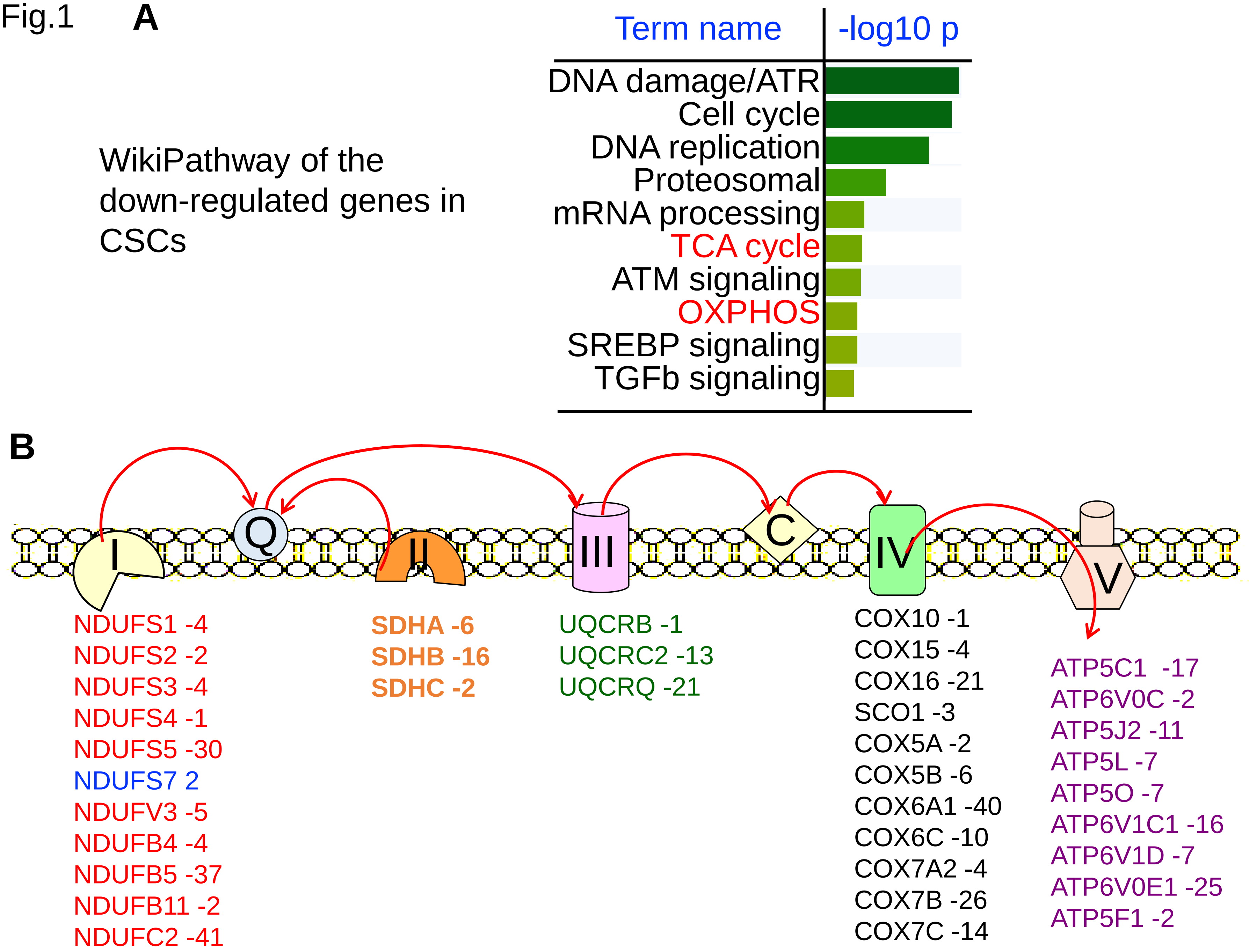
A
WikiPathway of the down-regulated genes in CSCs
Term name
-log10 p
DNA damage/ATR
Cell cycle
DNA replication
Proteosomal
mRNA processing
TCA cycle
ATM signaling
OXPHOS
SREBP signaling
TGFb signaling
B
I
Q
II
III
C
IV
V
NDUFS1 -4
NDUFS2 -2
NDUFS3 -4
NDUFS4 -1
NDUFS5 -30
NDUFS7 2
NDUFV3 -5
NDUFB4 -4
NDUFB5 -37
NDUFB11 -2
NDUFC2 -41
SDHA -6
SDHB -16
SDHC -2
UQCRB -1
UQCRC2 -13
UQCRQ -21
COX10 -1
COX15 -4
COX16 -21
SCO1 -3
COX5A -2
COX5B -6
COX6A1 -40
COX6C -10
COX7A2 -4
COX7B -26
COX7C -14
ATP5C1 -17
ATP6V0C -2
ATP5J2 -11
ATP5L -7
ATP5O -7
ATP6V1C1 -16
ATP6V1D -7
ATP6V0E1 -25
ATP5F1 -2

sFig. 2

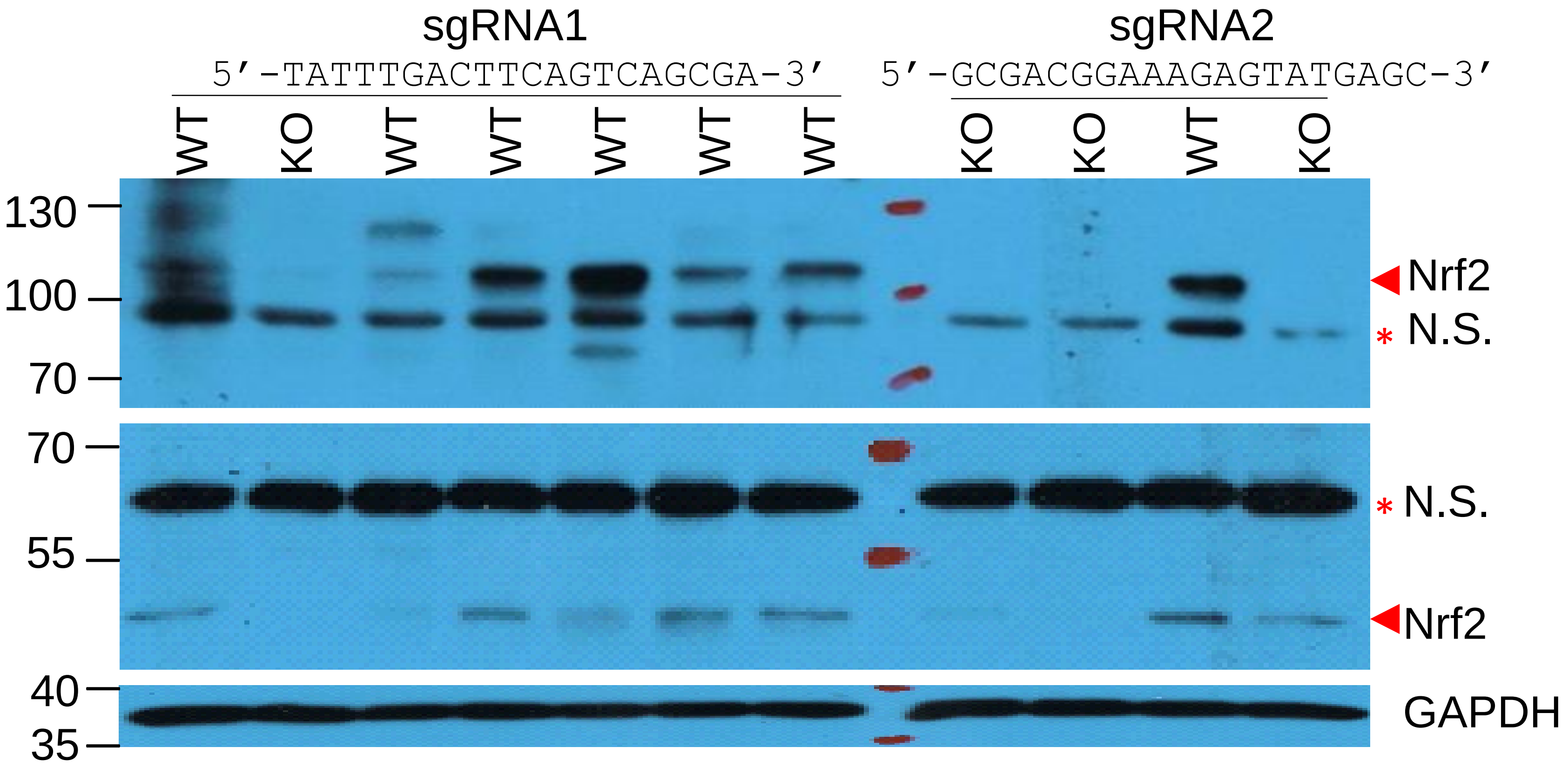

Sources of Nrf2 antibodies for screening:

Upper panel: 12721 Cell Signaling D1Z9C XP Rabbit mAb
Lower panel: sc-365949 A-10 Mouse mAb

sFig.3

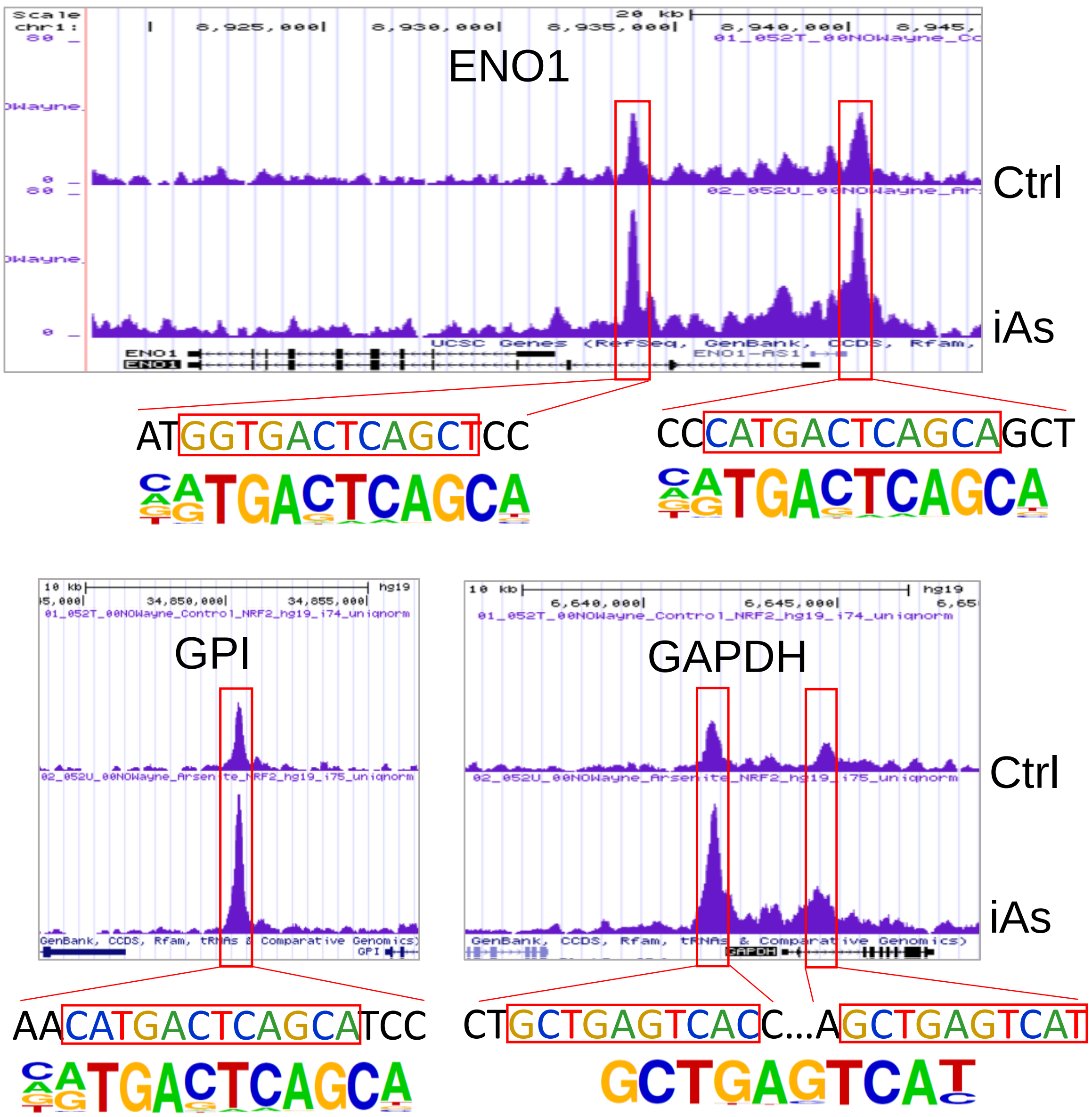

ENO1
Ctrl
iAs
ATGGTGACTCAGCTCC
CCCATGACTCAGCAGCT
GPI
GAPDH
Ctrl
iAs
AACATGACTCAGCATCC
CTGCTGAGTCACC...AGCTGAGTCAT

sFig. 4

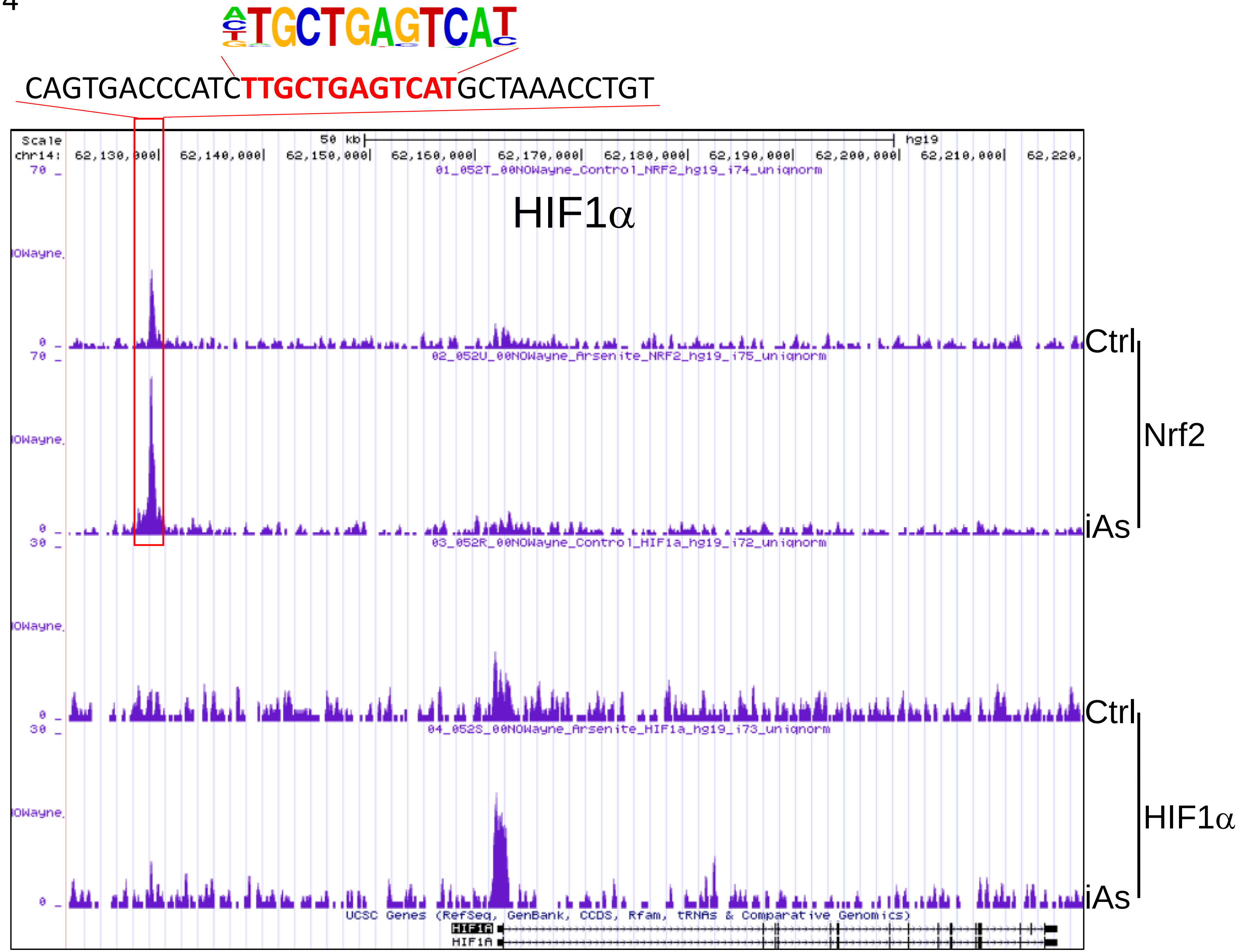

TTGCTGAGTCAT
CAGTGACCCATCTTGCTGAGTCATGCTAAACCTGT
Scale
50 kb
hg19
chr14: 62,130,000| 62,140,000| 62,150,000| 62,160,000| 62,170,000| 62,180,000| 62,190,000| 62,200,000| 62,210,000| 62,220,
01_052T_00NOWayne_Control_NRF2_hg19_i74_uniqnorm
HIF1α
02_052U_00NOWayne_Arsenite_NRF2_hg19_i75_uniqnorm
03_052R_00NOWayne_Control_HIF1a_hg19_i72_uniqnorm
04_052S_00NOWayne_Arsenite_HIF1a_hg19_i73_uniqnorm
UCSC Genes (RefSeq, GenBank, CCDS, Rfam, tRNAs & Comparative Genomics)
HIF1A
HIF1A
Ctrl
iAs
Nrf2
Ctrl
iAs
HIF1α

sFig. 5

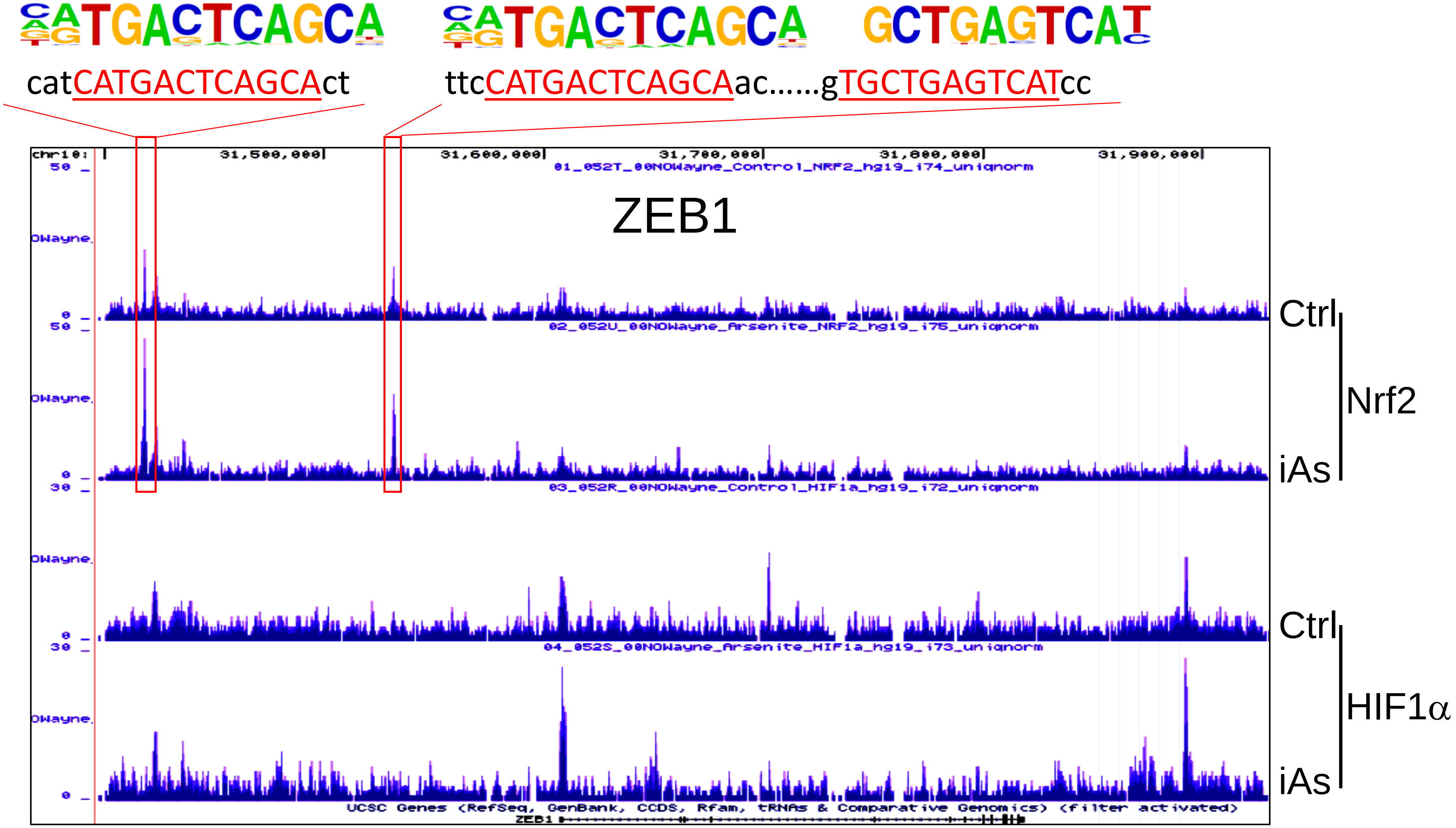
catCATGACTCAGCAct
ttcCATGACTCAGCAac……gTGCTGAGTCATcc
ZEB1
Ctrl
iAs
Nrf2
Ctrl
iAs
HIF1α

sFig. 6

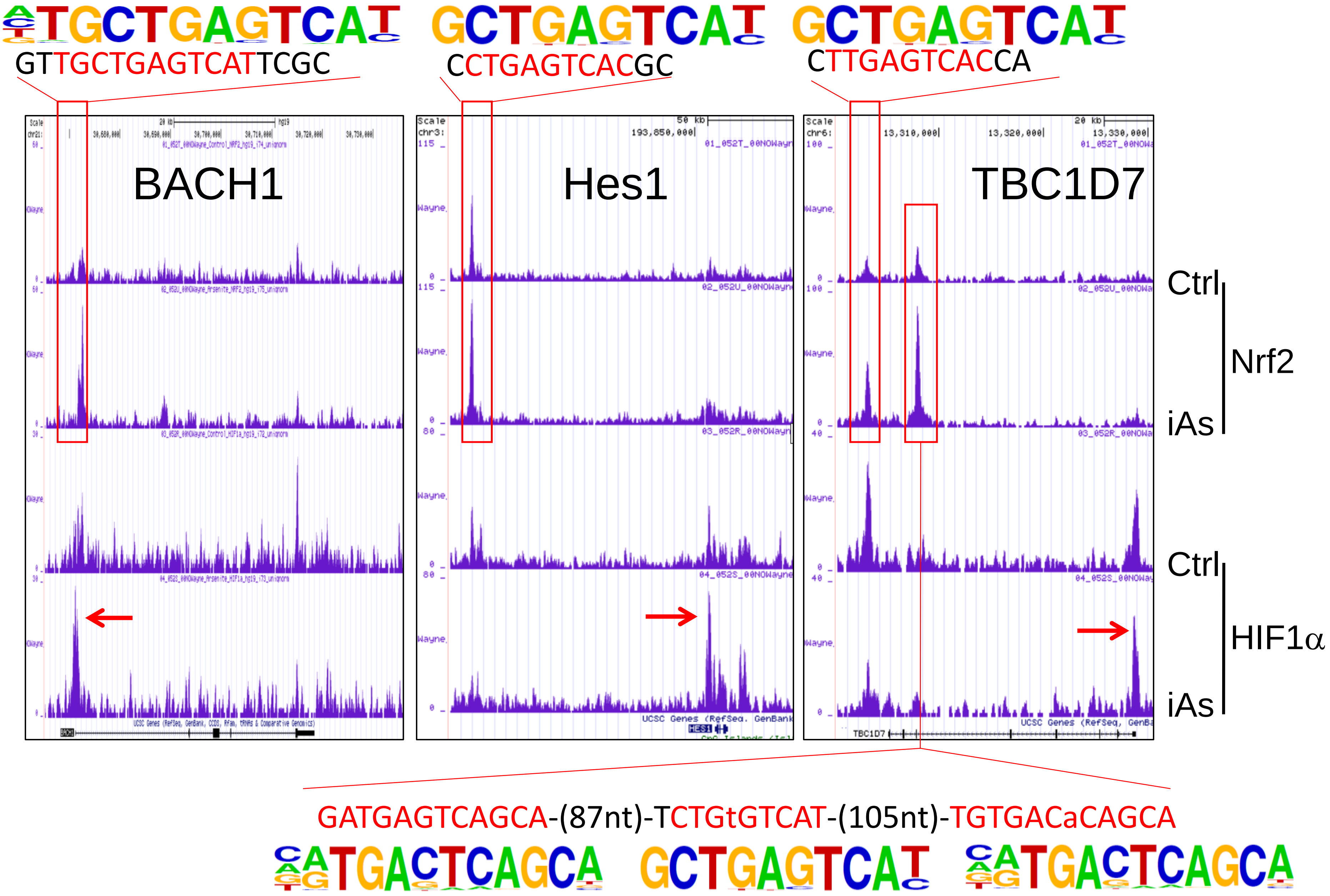

GTTGCTGAGTCATTCGC
CCTGAGTCACGC
CTTGAGTCACCA
BACH1
Hes1
TBC1D7
Ctrl
iAs
Nrf2
Ctrl
iAs
HIF1α
GATGAGTCAGCA-(87nt)-TCTGtGTCAT-(105nt)-TGTGACaCAGCA

sFig. 7

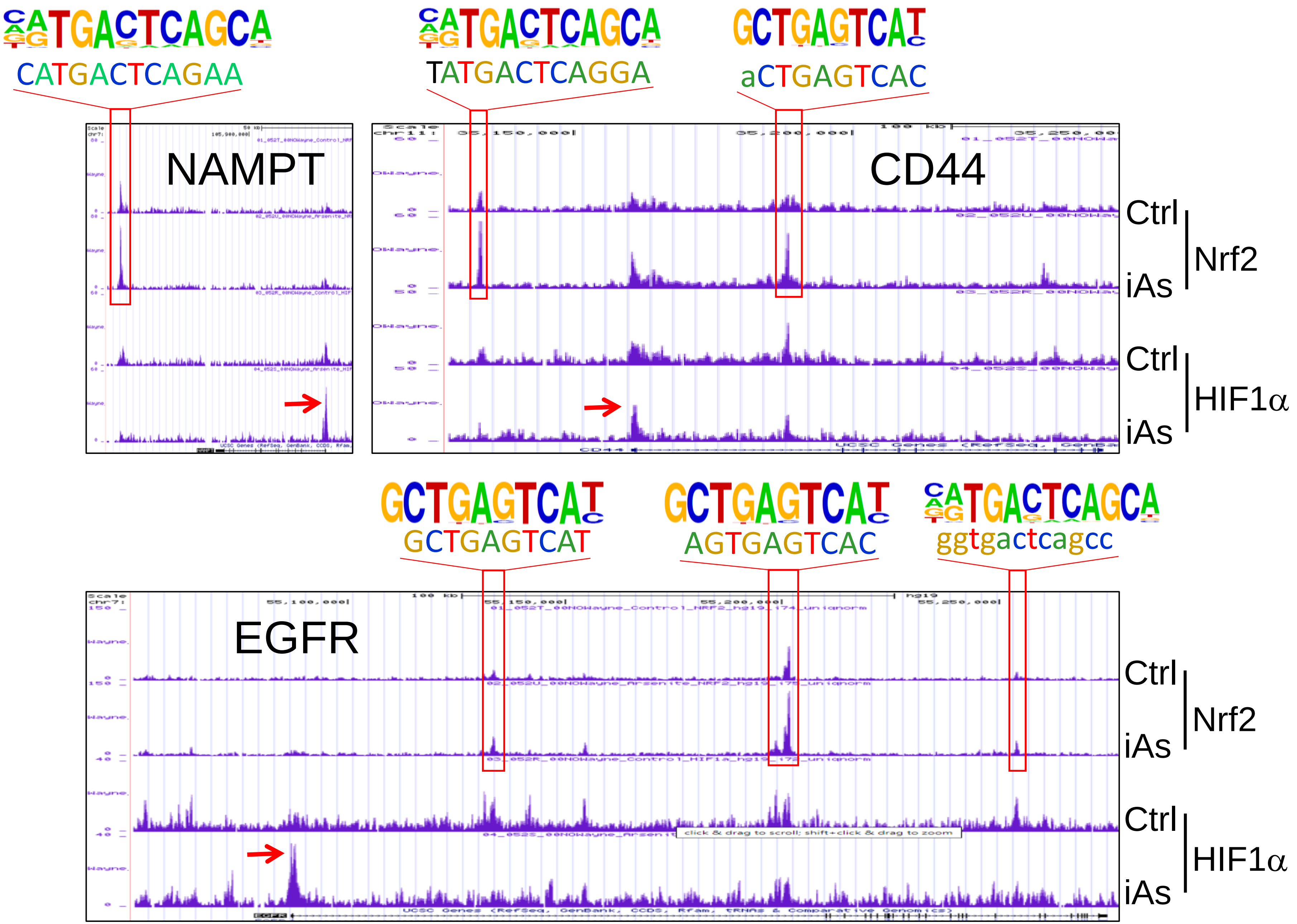
CATGACTCAGAA
TATGACTCAGGA
aCTGAGTCAC
NAMPT
CD44
Ctrl
iAs
Nrf2
Ctrl
iAs
HIF1α
GCTGAGTCAT
AGTGAGTCAC
ggtgactcagcc
EGFR
Ctrl
iAs
Nrf2
Ctrl
iAs
HIF1α
